# Transcriptome Analysis Suggested Striking Transition Around the End of Epiboly in the Gene Regulatory Network Downstream of the *Oct4*‐Type POU Gene in Zebrafish Embryos

**DOI:** 10.1111/dgd.70012

**Published:** 2025-06-09

**Authors:** Masaaki Ikeda, Kana Kobayashi, Yukiko Nakayama‐Sadakiyo, Yuto Sato, Ayano Tobita, Mika Saito, Kyo Yamasu

**Affiliations:** ^1^ Division of Life Science, Graduate School of Science and Engineering Saitama University Shimo‐Okubo, Sakura‐ku, Saitama City, Saitama Japan

**Keywords:** *Hes/her*, microarray, midbrain‐hindbrain boundary, *Oct4*/*pou5f3*, transcriptional regulation, zebrafish

## Abstract

Zebrafish *pou5f3* encodes a Class V POU transcription factor, Pou5f3, which regulates various developmental processes, including neurogenesis and brain formation. In the current study, we attempted to comprehensively identify the Pou5f3 downstream genes around the end of epiboly, when the competence of the mid‐hindbrain region to Pou5f3 suppression changes drastically, by the microarray method and a heat‐inducible dominant‐interference *pou5f3* gene (*en‐pou5f3*) that functionally suppresses *pou5f3*. At late epiboly and early somitogenesis stages, we identified genes whose expression was altered in *en‐pou5f3*‐induced embryos, revealing numerous genes regulated differently by Pou5f3 at the two stages. The validity of the microarray data was confirmed by whole mount in situ hybridization and quantitative RT‐PCR. Many of the downstream genes were implicated by the Gene ontology (GO) analyses in transcriptional regulation and neural development and were enriched with *sox* genes and bHLH genes such as *her* genes. Interestingly, we noticed a tendency that Notch‐dependent *her* genes were activated, whereas Notch‐independent *her* genes were downregulated by Pou5f3 suppression. Among the Notch‐independent *her* genes, *her3*, which is orthologous to mammalian *Hes3*, was suggested to be strongly activated endogenously by Pou5f3. In the upstream DNA of this gene, we found two noncoding conserved sequences (NCRs), which harbored consensus binding sites for Pou5f3, Sox, and Nanog. We further showed in reporter assays that the transcriptional regulatory activity of the *her3* upstream DNA was strongly enhanced by SoxB1, and this SoxB1‐mediated activation was weakened by Pou5f3. Deletion experiments showed that both upstream NCRs were involved in transcriptional repression.

## Graphical abstract

Transcriptome analysis of *pou5f3*‐regulated genes in zebrafish embryos showed that competence to *pou5f3* regulation changes significantly before and after the end of epiboly. The target genes encoded a diverse array of proteins, including transcriptional regulators and signaling molecules.

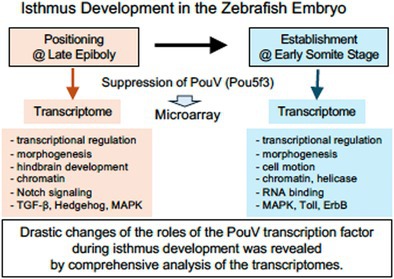

## 1 Introduction

The POU family of transcription factors is composed of six subfamilies (class I–VI), among which class V POU (PouV) factors are considered jawed vertebrate‐specific, and the major members are Pou5f1 and Pou5f3 (Bakhmet and Tomilin 2022; Onichtchouk 2016). Most mammals possess *pou5f1* as the major *PouV* gene; mouse *Pou5f1*, also called *Oct4*, plays pivotal roles in the pluripotency of early embryonic cells and embryonic stem cells (ESCs) (Loh et al. 2006; Ovitt and Schöler 1998).

Meanwhile, *pou5f3* is a *PouV* gene widely seen among nonmammalian vertebrates. In *Xenopus*, there are three *pou5f3* genes probably due to gene duplication that occurred during evolution, among which *pou5f3.3* is expressed maternally, whereas the remaining two are activated at the blastula stage. In mouse ESCs (mESCs), *Xenopus pou5f3* could substitute for the mouse orthologue for pluripotency (Morrison and Brickman 2006). Chicken *PouV* (*cPouV*; *pou5f3* orthologue) was shown to be effective in the maintenance of pluripotency and self‐renewal of mESCs. These observations make it likely that the functions of *PouV* in the maintenance of pluripotency are common among vertebrates (Lavial et al. 2007). Zebrafish *pou5f3* (previously called *pou2*) is maternally expressed throughout embryos, then confined to spotted patterns in the neural plate around the end of epiboly and restricted to the caudal‐most spinal cord during somitogenesis (Takeda et al. 1994; Yuikawa et al. 2021).

Analyses of *pou5f3* mutants (*spiel‐ohne grenzen/spg*, Schier et al. 1996) have revealed the pleiotropic functions of this gene; *pou5f3* is involved in dorsoventral patterning, endoderm differentiation, and gastrulation in early embryos (Lunde et al. 2004; Reim and Brand 2006; Reim et al. 2004; Song et al. 2013). Importantly, *pou5f3* is one of the major regulators of zygotic gene activation (ZGA) at the midblastula transition (MBT), which could account for the early defects in *spg* mutants (Bakhmet and Tomilin 2022; Leichsenring et al. 2013). At later stages, *pou5f3* is required for isthmus formation at the midbrain‐hindbrain boundary (MHB) as well as segmentation and neural development in the hindbrain (Belting et al. 2001; Burgess et al. 2002; Hauptmann et al. 2002; Reim and Brand 2002). Recently, alteration of the transcriptome was analyzed in maternal‐zygotic *pou5f3* mutants (*MZ‐spg*) from unfertilized eggs to mid‐gastrulation by the microarray analysis, identifying a variety of *pou5f3* downstream genes that extensively changed depending on the developmental stages (Onichtchouk et al. 2010), being compatible with the diverse roles of *pou5f3* during early development.

To further temporally dissect the diverse roles of *pou5f3* in embryos, we established a transgenic (Tg) fish line harboring a heat‐inducible dominant‐interference *pou5f3* gene (*en‐pou5f3*), which suppresses *pou5f3* functions at desired stages (Khan, Nakamoto, Okamoto, et al. 2012; Khan, Nakamoto, Tai, et al. 2012). By exploiting this Tg line, we confirmed the multiple roles of *pou5f3* suggested by mutant analyses (Inomata et al. 2020; Yuikawa et al. 2021); the two approaches of manipulating Pou5f3 functions resulted in essentially the same impacts on development, showing that the major effects of *en‐pou5f3* induction are functional suppression of endogenous Pou5f3.

Temporally regulated suppression further specified the developmental stages when Pou5f3 operates for different functions. Importantly, Pou5f3 suppression in late gastrulae (90% epiboly; 9 h post‐fertilization, 9 hpf) abrogated the isthmus, whereas the same treatment in early somitogenesis (3‐somite stage, 3‐ss; 11 hpf) severely deformed the isthmus (Khan, Nakamoto, Tai, et al. 2012), suggesting different roles of *pou5f3* in isthmus development before and after the end of gastrulation. Drastic changes in the roles of *pou5f3* were further shown regarding its regulation of MHB‐related genes (Maekawa et al. 2024). A striking finding was the marked shift in the subsequent mRNA expression dynamics after Pou5f3 suppression between these two stages, only 2 h apart. The expression of MHB‐forming genes was immediately downregulated and not restored later after *en‐pou5f3* induction at 90% epiboly; thus, the repression was considered permanent. In contrast, when *en‐pou5f3* was induced at 3‐ss, MHB gene expression was repressed similarly but then restored to normal levels: therefore, the repression was considered reversible. This impressive regulatory shift strongly suggested the occurrence of drastic changes between the two induction stages in terms of the regulation of MHB‐forming genes.

The MHB region and the isthmus pattern the midbrain and cerebellum, thus known as the isthmic organizer (Liu and Joyner 2001; Rhinn and Brand 2001). Here, neural differentiation is inhibited, resulting in the maintenance of an undifferentiated state (Geling et al. 2003). In general, neural differentiation is regulated positively and negatively by multiple regulatory pathways involving *soxB1* family genes and proneural genes, as well as various cellular interactions involving Notch signaling (Bertrand et al. 2002; Holmberg et al. 2008; Rogers et al. 2009; Schmidt et al. 2013; Beatus and Lendahl 1998; Weinmaster 1997; Louvi and Artavanis‐Tsakonas 2006). Among the *soxB1* genes, *sox1*, *sox2*, and *sox3* are widely activated in the induced neuroepithelium and drive neural specification. They also regulate the proliferation and differentiation of neural stem cells (NSCs) (Archer et al. 2011; Dee et al. 2008; Holmberg et al. 2008; Rogers et al. 2009; Uchikawa et al. 2011). Of note, *soxB1* genes suppress the functions of proneural genes, resulting in the maintenance of the immature neural progenitor cells (Bylund et al. 2003).

Interestingly, Sox2 and Oct4 cooperatively activate many pluripotency genes, such as *Fgf4*, *Nanog*, and *Oct4*, in mESCs (Ambrosetti et al. 2000; Kuroda et al. 2005). In zebrafish, SoxB1 factors work cooperatively with Pou5f3 to regulate ZGA and early embryogenesis (Okuda et al. 2010; Onichtchouk et al. 2010; Pálfy et al. 2020; Riesle et al. 2023; Veil et al. 2019; Leichsenring et al. 2013). We previously showed that Pou5f3 and Sox3 synergistically activate the transcription of *pou5f3*, forming a positive autoregulatory loop (Kobayashi et al. 2018).

In a traditional view, neural differentiation initially progresses within proneural clusters, where all cells are equivalent in terms of neural differentiation due to the expression of proneural genes. Once Delta‐Notch signaling is activated, *her/Hes* genes are induced and suppress neural differentiation and/or promote different fates by repressing proneural genes (Artavanis‐Tsakonas et al. 1999) (Mumm and Kopan 2000). Her/Hes proteins also repress *delta*, thus allowing adjacent cells to undergo neurogenesis. This process of lateral inhibition regulates the expression of proneural genes and *delta* genes and selects subsets of cells for neuronal differentiation. Some *her/Hes* genes are components of Notch signaling as noted above, whereas others suppress neural differentiation independent of Notch signaling (Katoh and Katoh 2007). In zebrafish, *her4.1* (*Hes5* orthologue) inhibits neuronal differentiation depending on Notch signaling (Stigloher et al. 2008), whereas the functions of *her3* (*Hes3* orthologue), *her6* (*Hes1*), *her5/her11* (*Hes7*), and *her9* (*Hes4*) are independent of Notch signaling and maintain neural progenitor pools (Bae et al. 2005; Geling et al. 2004; Hans, Scheer, et al. 2004; Ninkovic et al. 2005; Ohyanagi et al. 2025; Tsuruoka et al. 2025).

These findings together suggest that genetic networks involving *pou5f3*, *her* genes, and *soxB1* genes play pivotal roles in neurogenesis in the vertebrate neural plate. In this study, to reveal the dynamically changing gene network coordinating isthmic development and its associated neurogenesis around the end of gastrulation in zebrafish embryos, we sought to identify the downstream genes of *pou5f3* by the microarray approach immediately before and after the end of gastrulation, revealing both distinct and shared downstream genes for the two stages. Among the major regulated genes was *her3*, whose transcriptional regulation was further examined. The data obtained suggest that *pou5f3* regulates brain regionalization and neurogenesis in cooperation with *soxB1* and *her* genes.

## 2 Materials and Methods

### 2.1 Fish Husbandry and Maintenance

Adult zebrafish (*Danio rerio*, RW strain) were maintained at 26.5°C in a 14‐h light/10‐h dark cycle. Embryos were raised at 28.5°C until appropriate stages unless specified. Morphological features and/or hpf were used for staging embryos (Kimmel et al. 1995). All experiments using live fish complied with the protocols approved by the Committee for Animal Care and Use of Saitama University.

### 2.2 Temporally Controlled Inhibition of Endogenous *pou5f3* in Embryos Using a Heat‐Inducible Dominant‐Interference Gene

We employed a Tg fish line, harboring a chimaeric gene (*en‐pou5f3*) that encoded a fusion protein composed of the repressor domain of *Drosophila* Engrailed (EnR) and Pou5f3 and was under regulation by the heat shock promoter *Tg*(*hsp70l:Dme.En‐pou5f1* or *sud11Tg*; hereafter referred to as *hsp‐en‐pou5f3*), (Khan, Nakamoto, Okamoto, et al. 2012). Pou5f3 is considered a transcriptional activator in embryos (Khan, Nakamoto, Okamoto, et al. 2012; Parvin et al. 2008; Bakhmet and Tomilin 2022; Onichtchouk et al. 2010) and cultured cells (Kobayashi et al. 2018), enabling this chimeric gene to inhibit the function of endogenous *pou5f3* in embryos when exposed to heat shock treatment (Inomata et al. 2020; Khan, Nakamoto, Okamoto, et al. 2012).

For transient induction of *en‐pou5f3*, hemizygous *hsp‐en‐pou5f3* Tg fish (*en‐pou5f3* ^*+/−*^) were crossed with wild‐type fish, and the embryos thus obtained were treated at 37°C for 60 min (heat shock) and subjected to analysis. Fifty percent of the resulting embryos were expected to be hemizygous for *en‐pou5f3 (en‐pou5f3^+/−^*), and the remaining should be wild‐type (*en‐pou5f3^−/−^*).

### 2.3 Genotyping of Embryos

To discriminate between *en‐pou5f3* ^*+/−*^ embryos and *en‐pou5f3* ^+/+^ embryos (wild‐type siblings), genomic DNA was extracted separately from individual embryos (live or stained), and the presence of the *en‐pou5f3* sequence was examined by polymerase chain reaction (PCR) using the extracted DNA as templates (Khan, Nakamoto, Okamoto, et al. 2012; Nakayama et al. 2013).

### 2.4 Extraction of Genomic DNA and RNA from Single Tg Embryos

Genomic DNA and total RNA were separately purified from individual embryos after heat shock using Isogen (Nippon Gene, Tokyo, Japan) according to the manufacturer's protocol. Respective heat‐treated embryos were genotyped as either *en‐pou5f3* ^*+/−*^ or *en‐pou5f3* ^*−/−*^ (wild‐type), as described above. RNA from individual embryos was pooled by the genotype and subjected to further analyses (Nakayama et al. 2017).

### 2.5 Quantitative Reverse‐Transcription PCR Analysis

RNA pools from *en‐pou5f3*‐induced and sibling embryos were used as templates for reverse transcription with random primers (hexa‐deoxyribonucleotide mixture, Takara) using M‐MLV Reverse Transcriptase (Invitrogen). Quantitative RT‐PCR (qRT‐PCR) was conducted with the synthesized cDNA using the Thunderbird SYBR qPCR Mix (Toyobo) and the StepOnePlus Real‐Time PCR System (Applied Biosystems). Primer pairs for respective genes of interest were designed using NCBI Primer‐BLAST (http://www.ncbi.nlm.nih.gov/tools/primer‐blast/; Table S1).

### 2.6 In Situ Hybridization

RNA probes labeled with digoxigenin (DIG) /fluorescein (FLU) were synthesized with the DIG/Fluorescein RNA Labeling Mix (Roche Diagnostics) using T3 or T7 RNA polymerase (Stratagene), according to the manufacturers' protocols. Single‐ and two‐color whole mount in situ hybridization (WISH) was conducted as described previously (Jowett 2001; Kikuta et al. 2003).

### 2.7 Plasmid Constructs for Luciferase Assay

For the luciferase assay, the 4.0‐kb upstream region of zebrafish *her3* (cf. Figure S2), which was previously shown to include the closely adjacent binding sites for Sox and POU (Okuda et al. 2010; Onichtchouk et al. 2010), was ligated into the multicloning site (MCS) of pGL4.10[luc2], in which modified firefly luciferase, luc2, is employed as a reporter (referred to as pGL4 hereafter, Promega) (pHer3[−4.0]‐Luc, cf. Figure 5D). The same upstream 4.0‐kb DNA was inserted into the MCS of pEGFP‐1 (pHer3[−4.0]‐EGFP) for in vivo reporter experiments. Deletion of noncoding conserved regions (NCRs) 1 and/or 2 was conducted from pHer3[−4.0]‐Luc by inverse PCR (pHer3[−4.0]dNCR1‐Luc, pHer3[−4.0]dNCR2‐Luc), pHer3[−4.0]dNCRs‐Luc) (Text S1).

The coding regions of regulatory genes were amplified by reverse‐transcription PCR of total RNA from embryos and ligated into pCS2+ (Table S2), which were used as effector genes in luciferase assays. The expression of the genes was driven in transfected cells by the upstream cytomegalovirus promoter. For control, pCS2+ harboring the *enhanced green fluorescent protein* (*egfp*) gene (pCS2+egfp) was used instead.

For synthesis of *en‐pou5f3*‐ERT2 mRNA, the template plasmid was built; the detail will be available on request (cf. Figure 4A). Briefly, the coding sequence of *en‐pou5f3* lacking the stop codon was amplified by PCR and replaced with the KalTA4 sequence in pKalTA4‐ERT2 (kindly donated by Dr. M. Tada), resulting in a pCS2+ −based plasmid, in which the complete *en‐pou5f3* coding sequence was fused to the ERT2 sequence (pCS2+en‐pou5f3‐ERT2).

PCR for plasmid construction was conducted with high‐fidelity DNA polymerase (LA Taq polymerase, TaKaRa), and the structures of the constructs were verified by sequencing.

### 2.8 Luciferase Assay Using Cultured Cells

HEK293T and P19C6 (a subclone of the mouse embryonal carcinoma (EC) cell line, P19; hereafter referred to as P19; Gao et al. 2001) cells were used for luciferase assays. Cell culture and transfection of the reporter and effector constructs were conducted as previously described (Kobayashi et al. 2018). pGL4.75 harboring the *Renilla* luciferase gene (Promega) was used as an endogenous control, and pCS2+ egfp was used as a control effector. Total amounts of transfected DNA were adjusted with pCS2+ DNA. Cells were subjected to luciferase assays using the Dual Luciferase Reporter Assay System (Promega). Three wells were used for each determination, and firefly luciferase (luc2) activity was standardized to that of *Renilla* luciferase. Statistical significance was examined by a two‐sample *t*‐test (cf. Figures 2, 7, 8). Reporter assays were repeated at least three times to confirm reproducibility. For in vitro neural induction, P19 cells were administered with 1.0 × 10^−6^ retinoic acid (RA) 8 h after transfection with reporters and effectors and further cultured (cf. Figure 7A).

To know the expression levels of reporters at the mRNA level, HEK293T cells were transfected with plasmids as above, total RNA was extracted from cells in each well (triplicates), and the expression levels of luc2 in addition to *Renilla* luciferase as an internal control were quantified by qRT‐PCR, using the primer pairs specific to the reporter genes (Table S1). The experiments were repeated three times to confirm reproducibility, and the *t*‐tests were conducted as well.

### 2.9 Microarray Analysis

Embryos from crosses between *en‐pou5f3* ^*+/−*^ and wild‐type fish were subjected at 90% epiboly or 3‐ss to heat shock as described above. After a 30 min interval at normal culture temperature, genomic DNA and total RNA were separately extracted from 10 to 12 individual embryos, and respective heat‐treated embryos were genotyped using the genomic DNA. RNA was purified from the pooled extracts combined based on the genotype (> 5 embryos/pool).

Purified RNA was subjected to the microarray analysis (KURABO, Osaka, Japan) using the Affymetrix GeneChip Zebrafish Genome Array (15,617 probes, Affymetrix, Santa Clara, CA, USA) to comprehensively compare the transcriptome between *en‐pou5f3* ^*+/−*^ and wild‐type embryos (hereafter referred to as ‘*en‐pou5f3+*’ and ‘sibling’ embryos, respectively) employing the single comparison analysis following the protocol provided by Agilent, as was conducted before (Nakayama et al. 2017) (Table S3) (see Text S1 for details). It should be mentioned that the term ‘probe’ is correct, but for convenience's sake, the word ‘gene’ will be frequently used instead hereafter. The Affymetrix GeneChip Operating Software (GCOS) was used to determine signal intensities and detection calls for each gene. Alteration of the expression levels was tested by the Wilcoxon signed‐rank test. When more severe criteria were necessary, the data were extracted when the fold change is more than twice or less than half. Gene ontology (GO) analysis (Aleksander et al. 2023), Pathway analysis (García‐Campos et al. 2015), and InterPro analysis (Paysan‐Lafosse et al. 2023) were performed based on these data using DAVID Bioinformatics Resources 6.7 (https://david.ncifcrf.gov) for GO analysis, Pathway analysis, and InterPro analysis and DAVID 6.8 (https://davidbioinformatics.nih.gov/summary.jsp) for Functional Annotation Clustering analysis (Sherman et al. 2022). The significance of the association was examined by the Fisher's exact test. The primary microarray data generated in this study have been submitted to GEO (http://www.ncbi.nlm.nih.gov/geo/) (GSE271502, GSM8378058, GSM8378059, GSM8378060, GSM8378061).

### 2.10 Microinjection Experiments

For synthesis of capped mRNA, template plasmids were linearized and transcribed using the MEGAscript SP6 Kit (Ambion) according to the manufacturer's protocol. Synthesized mRNA was pressure‐injected into single blastomeres of one‐cell to four‐cell stage embryos. *egfp* mRNA was injected into embryos as a negative control. For in vivo reporter assays, EGFP constructs were injected similarly at 5 pg/embryo.

### 2.11 Microscopy Observation

The images of embryos were captured using a fluorescence stereomicroscope (Leica, MZFLIII) and a CCD camera (Pengin600CL, Leica, DFC300FX). Fluorescence views were observed using a GFP filter. Observation of cultured cells was performed under an inverted microscope (ECLIPSE Ti, Nikon), Intensilight C‐HGFIE (Nikon), DS‐U3 (Nikon), and a GFP positive filter.

### 2.12 Sequence Analyses

The genomic sequences (zebrafish, ENSDARG00000076857; spotted gar, ENSLOCG00000005161) and exon sequences (Transcript ID; zebrafish, ENSDART00000113803.3; spotted gar, ENSLOCT00000006224.1) of *her3* were obtained from Ensembl Genome Data Resources (Sanger Center/Wellcome Trust; http://www.ensembl.org/index.html) (Martin et al. 2023). Sequence comparison and prediction of the binding sites for three transcription factors (Oct, Sox, Nanog) were conducted using rVISTA (http://genome.lbl.gov/vista/index.shtml) (Loots et al. 2002). Alternatively, transcription factor binding sites were predicted by Match‐1.0 Public (http://www.gene‐regulation.com/pub/databases.html).

### 2.13 Activation of En‐Pou5f3 in Embryos by the Tamoxifen‐ERT2 System

*en‐pou5f3*‐ERT2 mRNA was injected into embryos (150 pg/embryo), which were allowed to develop until 50%–70% epiboly, dechorionated, and washed four times with E3 solution. Embryos were then transferred to wells of a 24‐well plate (Thermo, 144,530) (~5 embryos/500 μL E3/well) whose bottom had been covered with 1% agarose in 1/3 Ringer solution and were further allowed to develop. These embryos were treated with E3 solution containing 5 μM 4‐hydroxytamoxifen (4‐OHT, SIGMA‐ALDRICH) and incubated for two more hours, and then fixed with 4% paraformaldehyde for WISH analyses.

To inhibit protein synthesis during 4‐OHT treatment, cycloheximide (CHX, WAKO) was added to culture medium (50 μg/mL) 30 min in advance. As CHX treatment delayed development, fixation of embryos was performed at either of two timings: at the same chronological timing (age matching) or at the same morphological stage (stage matching).

## 3 Results

### 3.1 Comprehensive Analysis of the Downstream Genes of *pou5f3*

During zebrafish embryogenesis, epiboly movement—the main morphogenetic movement in gastrulation—ends at 10 hpf, after which somitogenesis and axis elongation take place. Notably, the effects of *en‐pou5f3* induction on isthmus formation differ markedly between the late epiboly stage (e.g., 90% epiboly) and early somite stages (e.g., 3‐ss) (Khan, Nakamoto, Tai, et al. 2012), strongly suggesting that *pou5f3* controls distinct aspects of MHB development at these stages (Maekawa et al. 2024). Specifically, Pou5f3 suppression at 90% epiboly abrogated the isthmus structure, whereas suppression at the early somite stage led to severe deformation of the isthmus. In addition, significant changes were observed in the regulation of MHB‐related genes depending on the timing of *pou5f3* suppression, suggesting the occurrence of drastic changes during the short period of 2 h in terms of the regulatory mechanism of MHB‐forming genes. Previous transcriptome analysis using *MZ‐spg* embryos identified *pou5f3* downstream genes in early embryos up to the mid‐gastrula stage (0–8 hpf) (Onichtchouk et al. 2010). However, this study did not address downstream genes around the end of epiboly. Furthermore, the transcriptome of *MZ‐spg* embryos reflects the combined loss of both maternal and zygotic Pou5f3, making it difficult to dissect temporal effects.

Thus, we undertook new transcriptome analyses to comprehensively identify the target genes of *pou5f3* specifically at 90% epiboly and 3‐ss. Embryos derived from crosses of *en‐pou5f3* ^*+/−*^ fish and wild‐type fish were exposed to heat shock at either of these two stages, then allowed to develop for an additional 30 min to permit mRNA maturation. Individual treated embryos were subsequently subjected to extraction of DNA and RNA. Each embryo was genotyped using extracted DNA, and RNA samples were pooled by genotypes for both stages.

Using these RNA preparations as templates, microarray analyses were conducted to capture the global transcriptomic changes induced by *en‐pou5f3* at each stage. GCOS software was used to determine signal intensities and detection calls (cutoff *p*‐values; α1 = 0.05, α2 = 0.065, Tau = 0.015). Alteration of the expression levels was assessed by the Wilcoxon signed‐rank test, with statistical significance defined as *p* < 0.0025 for upregulation and *p* > 0.9975 for downregulation (Table S3).

**FIGURE 1:**
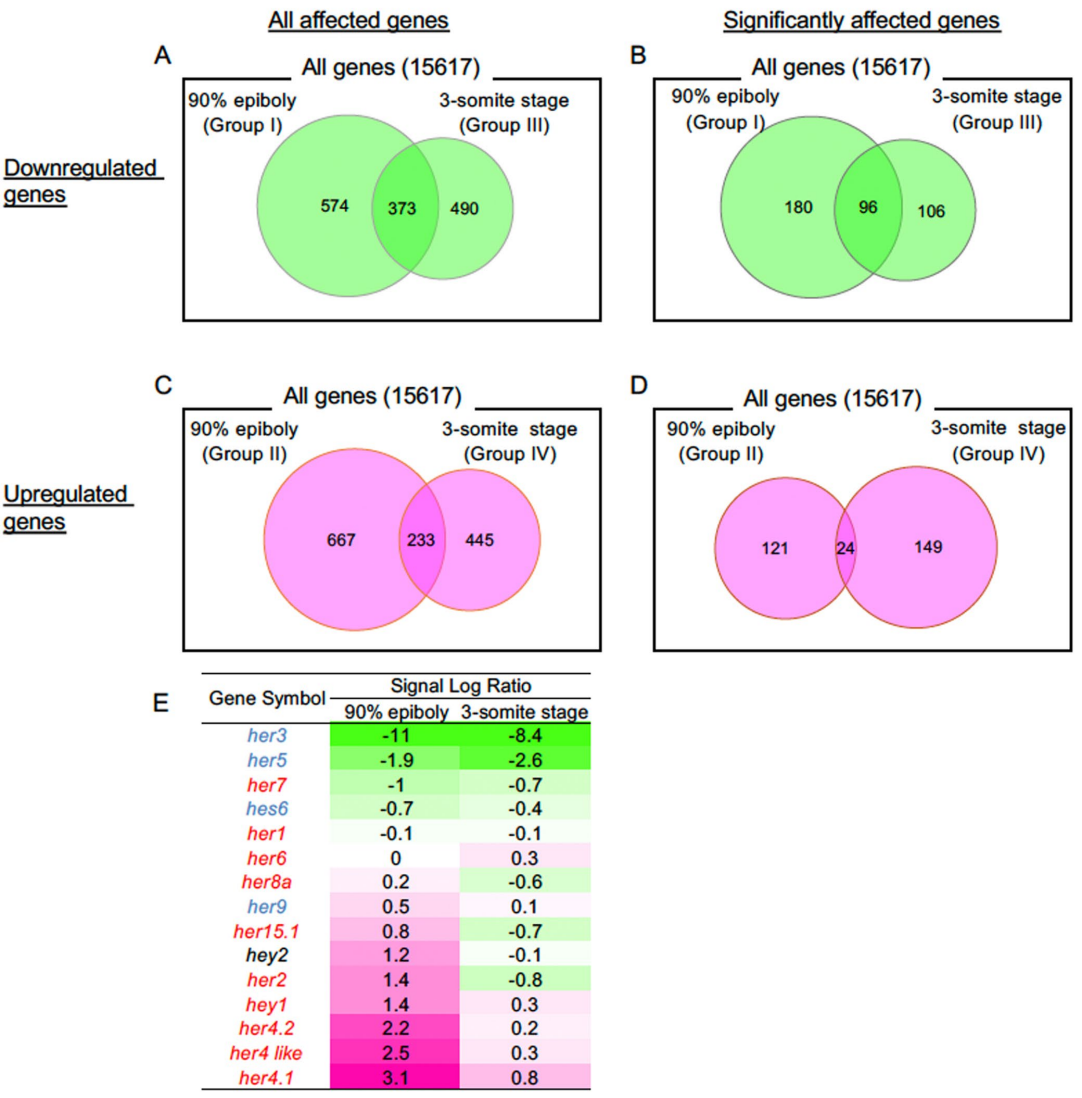
Microarray analysis of the altered transcriptome due to suppression of endogenous *pou5f3* in zebrafish embryos. (A–D) Venn diagrams showing the numbers of genes/probes (total number, 15,617) whose expression was downregulated (A, B) or upregulated (C, D) by *en‐pou5f3* induction at 90% epiboly (left) or 3‐ss (right), respectively. (A, C) All genes whose expression was judged to have been significantly affected by the Wilcoxon signed‐rank test (upregulated, *p* < 0.0025; downregulated, *p* > 0.9975). (B, D) Genes whose expression was evidently affected by *en‐pou5f3* induction by twice or more (signal log ratio, > 1 or < −1). E. Alteration of *bHLH‐O* gene expression due to *en‐pou5f3* induction. Signal log ratios showing changes in expression levels, obtained by the microarray, are shown with a heat map for the two induction stages. The genes are arranged based on the signal log ratios at 90% epiboly. Green indicates downregulation, and magenta indicates upregulation. Blue letters indicate *her*‐related genes that function in a Notch‐independent manner, whereas the genes shown in red are Notch‐dependent.

What was striking regarding the obtained data was that numerous genes were upregulated or downregulated by *en‐pou5f3* induction at both developmental stages. Specifically, *en‐pou5f3* induction at 90% epiboly downregulated 947 genes (Group I) and upregulated 900 genes (Group II). Meanwhile, induction at 3‐ss decreased the expression levels of 863 genes (Group III), but the same treatment increased the expression of 678 genes (Group IV) (Figure 1A,C). Among these affected genes, 373 genes were downregulated, whereas 233 genes were upregulated at both stages. Meanwhile, we identified stage‐specific *en‐pou5f3*‐regulated genes as well. Regarding downregulation, 574 genes were identified at 90% epiboly alone, while the expression of 490 genes was decreased specifically at 3‐ss. In contrast, 667 genes were upregulated specifically at 90% epiboly, whereas 445 genes were identified at 3‐ss alone. Instances of contradictory regulation were rare: 25 genes were downregulated at 90% epiboly but upregulated at 3‐ss, whereas 39 genes exhibited the opposite pattern (data not shown).

We further compared the transcript profiles using a more stringent criterion of a greater than two‐fold change (Figure 1B,D), showing that *en‐pou5f3* induction at 90% epiboly downregulated 276 genes (Table S4) and upregulated 145 genes (Table S5). The same treatment at 3‐ss lowered the expression levels of 202 genes (Table S6) and increased the expression of 173 genes (Table S7). Among these affected genes, 96 genes were downregulated (Table S8), whereas 24 genes were upregulated (Table S9) at both stages. With regard to stage‐specific regulation, 180 genes were downregulated at 90% epiboly alone (Table S10), while 106 genes were repressed specifically at 3‐ss (Table S11). In contrast, 121 genes were upregulated specifically at 90% epiboly (Table S12), whereas 149 genes were identified as upregulated at 3‐ss alone (Table S13). These findings indicate that *pou5f3*‐downstream genes are largely different between 90% epiboly and 3‐ss, with only small intersections.

As *en‐pou5f3* suppresses the function of the endogenous gene in a dominant‐negative manner, it is likely that downregulated genes are positively regulated, and upregulated genes are negatively regulated, by endogenous Pou5f3. Only a minor fraction of genes is regulated in reverse directions at each of the two stages. At 90% epiboly, the most significantly downregulated genes were *her3, pax6b, otx1a, hesx1, foxb1a*, and *irx1a*, whereas the most strikingly upregulated genes were *sox9b, lect1*, *pou3f3a*, *slc20a1a*, *hhatlb*, and *pik3r1* (Table 1A,B). Of note, these upregulated genes were followed by genes involved in MHB/hindbrain development, such as *gbx2, egr2b*, and *her4.1* (Table S4). Meanwhile, at 3‐ss, the most strikingly downregulated genes were *her3, sox21b, zic4, lrrtm1, barhl2*, and *six7*, whereas the most significantly upregulated genes were *pth2r* (*parathyroid hormone 2 receptor*), *kif5aa* (*kinesin family member 5A, a*), *sebox, stxbp6l* (*syntaxin binding protein 6* (*amisyn*), *like*), *cops4* (*COP9 constitutive photomorphogenic homolog subunit 4* (*Arabidopsis*)), and *slc16a9a* (Table 1C,D). *her3* was notably downregulated at both stages. Interestingly, genes significantly downregulated (Group I/III) or upregulated (Group II) included brain regionalization genes (I, *pax6b, otx1a, hesx1, foxb1a, irx1a*; II, *gbx2*, *egr2b*; III, *zic4*) or neurogenesis‐regulating genes (I, *her3*; II, *sox9b*, *pou3f3a*, *her4.1*; III, *lrrtm1*, *barhl2*), whereas genes upregulated at 3‐ss (Group IV) are known to be involved in the functions of mature neural cells.

**TABLE 1:** Genes that showed the most significant alterations in expression due to *en‐pou5f3* induction.^a^

A. Downregulated at 90% epiboly (Group I)					B. Upregulated at 90% epiboly (Group II)				
Gene	Signal Log Ratio^b^	*p* ^c^	*MZ‐spg* ^d^	Pou5f3 sites^e^	Gene	Signal Log Ratio^b^	*p* ^c^	*MZ‐spg* ^*d*^	Pou5f3 sites^e^
*her3*	−11.0	1	A	3/3	*sox9b*	4.5	0	—	0/0
*pax6b*	−6.2	0.999993	—	1/1	*lect1*	4.4	0.000408	—	0/0
*otx1a*	−5.1	0.999999	—	1/2	*slc20a1a*	4.3	0.001304	—	0/0
*hesx1*	−5.1	0.999999	—	1/1	*pou3f3a*	4.2	0.001991	—	0/1
*foxb1a* (*foxb1.2*)	−5.0	1	A	1/3	*hhatlb*	3.5	0.000357	—	0/0
*irx1a*	−4.8	1	—	0/0	*pik3r1*	3.3	0.000205	—	0/2
					*tbx2b* ^*f*^	2.9	0	DE	0/0
					*gbx2* ^f^	2.5	0	DE	1/0
					*egr2b* ^f^	2.5	0	—	0/0
					*her4.1/her4.2* ^f^	2.5	0	—	0/0

C. Downregulated genes at 3‐ss (Group III)					D. Upregulated genes at 3‐ss (Group IV)				
Gene	Signal Log Ratio^b^	*p* ^c^	*MZ‐spg* ^d^	Pou5f3 sites^e^	Gene	Signal Log Ratio^b^	*p* ^c^	*MZ‐spg* ^d^	Pou5f3 sites^e^
*her3*	−8.4	1	A	3/3	*pth2r*	5.1	0.000002	—	0/0
*sox21b*	−4.9	0.999999	DE	0/0	*kif5a*	4.5	0	—	0/0
*zic4*	−4.8	0.999992	—	1/0	*sebox*	3	0.001876	—	0/0
*lrrtm1*	−4.0	0.999795	—	1/0	*stxbp6l*	3	0.000955	—	0/0
*barhl2*	−3.9	0.999966	—	2/0	*cops4*	2.8	0.000001	—	0/0
*six7*	−3.6	0.999999	A	0/1	*slc16a9a*	2.6	0.000004	—	0/0

^a^ Top six genes are listed in each Group showing the most significant alterations in expression levels due to *en‐pou5f3* induction.

^b^ Signal log ratios represent the logarithms of the ratios of *en‐pou5f3*‐induced expressions to controls.

^c^ The GCOS software was used to determine signal intensities and detection calls for each gene (cutoff *p*‐values; α1 = 0.05, α2 = 0.065, Tau = 0.015). Alteration of the expression levels was tested by the Wilcoxon signed‐rank test and judged to be statistically significant (upregulated, *p* < 0.0025; downregulated, *p* > 0.9975).

^d^ Categories reported in the microarray analyses on *MZ‐spg* mutants where *pou5f3* were homozygous both maternally and zygotically (Onichtchouk et al. 2010). A, downregulated at 8 hpf in *MZ‐spg* mutants; D/E, upregulated at 8 hpf in *MZ‐spg* mutants.

^e^ Numbers of Pou5f3 binding sites previously identified by ChIP‐Seq within 20 kb upstream of the transcription start site at the pre‐MBT stage (512‐cell stage) and post‐MBT stage (5 hpf) for respective genes (pre‐ MBT/post‐MBT) (Leichsenring et al. 2013).

^f^ In the case of Group II, genes involved in brain regionalization showed significant downregulation and are additionally shown.

Comparison of the transcriptomes between our study (*pou5f3* suppression around the bud stage) and the previous study using *MZ‐spg* mutants at 8 hpf (Onichtchouk et al. 2010), the latest stage examined (Table 1, Tables [Link], [Link]; category A, downregulation; category DE, increase), showed that the expression of a limited number of genes was altered in similar ways despite the significant difference in the stages examined (late gastrula and 3‐ss vs. mid‐gastrulae, respectively) (downregulation, 14 out of 90 genes at 90% epiboly and 9 out of 51 genes at 3‐ss; Tables S4 and 6; upregulation, 2 out 35 genes at 90% epiboly and 0 our of 40 genes at 3‐ss; Tables [Link], [Link]). This was also the case among significantly affected genes (Table 1; Group I, *her3, foxb1a*; Group II, *tbx2b, gbx2*; Group III, *her3, six7*; Group IV, *sebox, slc16a9a*; cf. Tables [Link], [Link]). These data reinforce the validity of our dominant‐interference approach on one hand and have also revealed the stage‐specific functions of *pou5f3* on the other hand. Interestingly, in all cases, *her3* was the significantly downregulated gene. In *MZ‐spg* mutants, *her3* showed decreased expression even from 4 hpf. These results strongly suggest that this *her* gene plays pivotal roles downstream of *pou5f3* throughout early development. Notably, other *her* genes were also regulated by *en‐pou5f3* as described below.

### 3.2 Confirmation of the Validity of the Microarray Data

**FIGURE 2:**
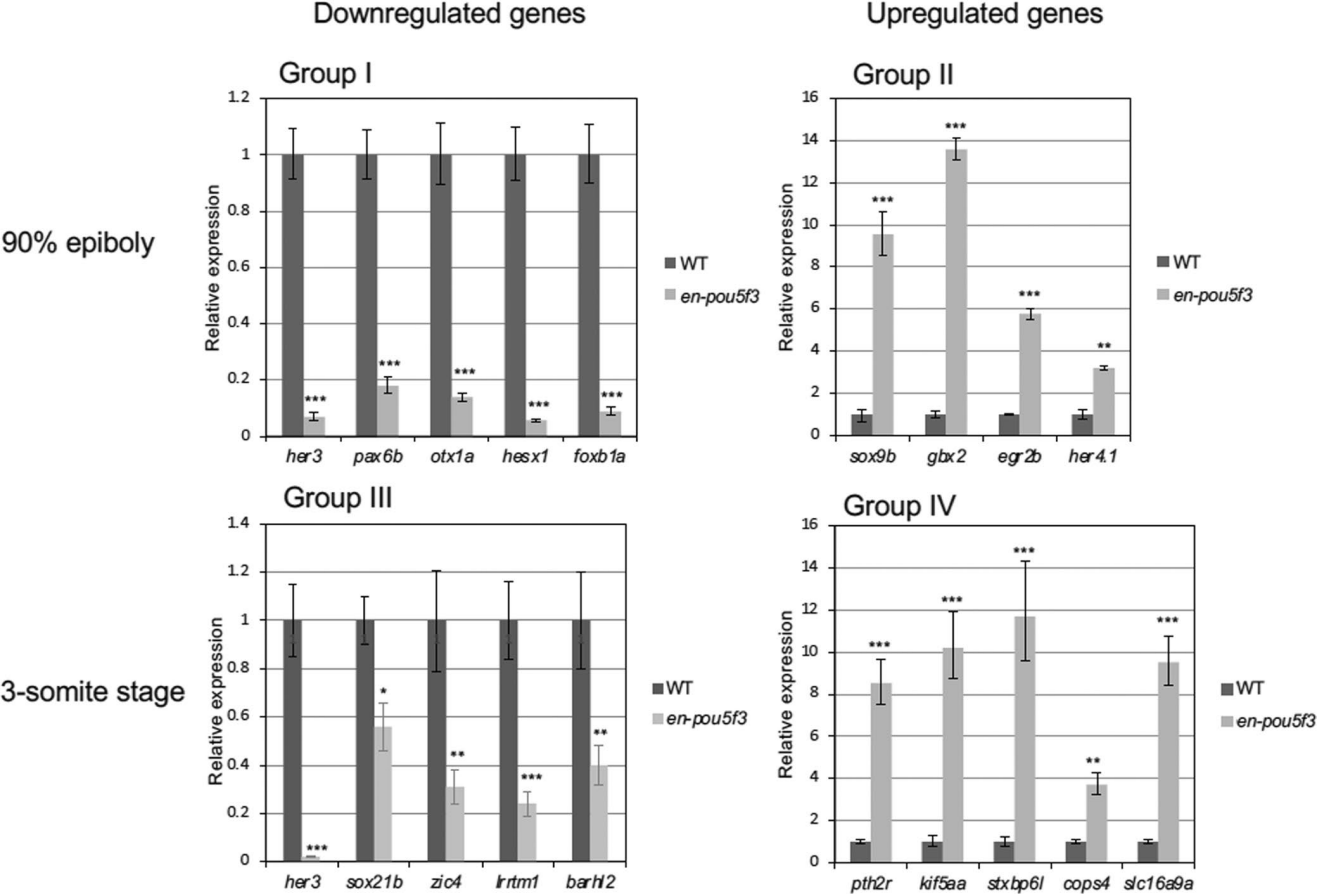
Quantitative analysis of the expression of genes that were shown to markedly change in the expression level by the microarray analyses. Expression was reevaluated by qRT‐PCR to confirm the validity of the microarray data for the genes that showed marked alteration in the expression level (Group I–IV) and/or those involved in brain formation (Group II) (Table 1). Regarding several Group II genes and *sebox* that had shown strikingly altered expression in the microarray, we failed to reliably quantitate the expression for unknown reasons and not shown here. The experiments were repeated three times, resulting in essentially the same results. The ordinates represent the expression levels in the *en‐pou5f3*‐induced embryos (light gray bars) relative to those in wild‐type sibling embryos (dark gray bars) for respective genes. Error bars, standard errors of means. *, *p* < 0.05; **, *p* < 0.01; ***, *p* < 0.001.

To confirm the validity of the microarray data, RNA pools from either *en‐pou5f3* ^+/−^ or wild‐type embryos, which had been exposed to heat shock at 90% epiboly or 3‐ss, were used in qRT‐PCR to quantify the expression of genes (Figure 2) that were significantly altered according to the microarray results (Table 1). In the case of some Group II genes, the data were unreliable, probably due to low expression levels; for these, the data for brain‐forming genes (*gbx2, egr2b, her4.1*) are shown instead (Figure 2). As a result, the microarray data were quantitatively confirmed for all genes examined. Essentially the same results were observed previously for the downregulation of *her3* and the upregulation of *gbx2* and *her4.1* at 90% epiboly, supporting the present data (Inomata et al. 2020).

**FIGURE 3:**
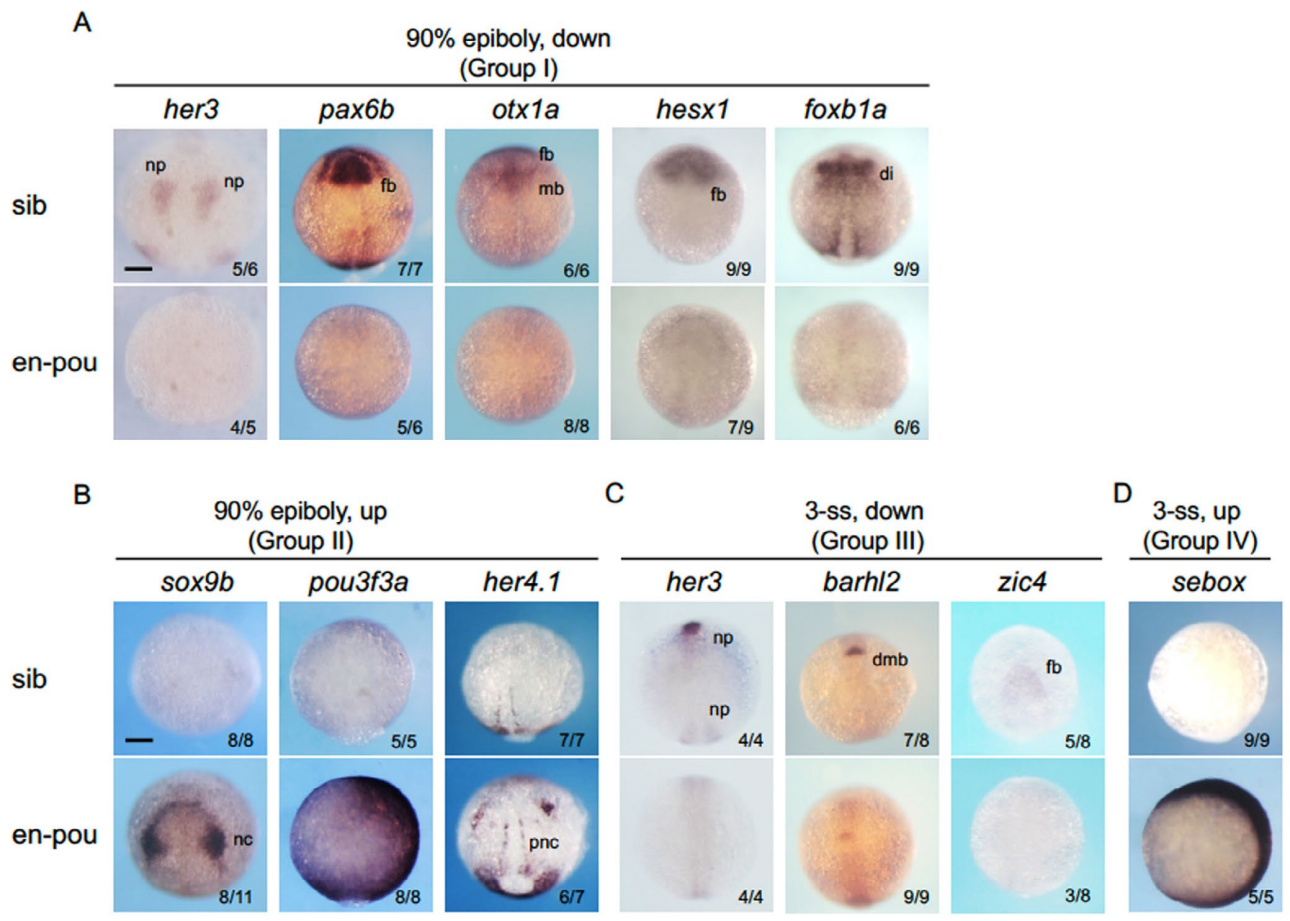
Confirmation of the altered gene expression by WISH in *en‐pou5f3*‐induced embryos. The expression patterns of the genes that had been shown to markedly differ in expression levels by the microarray analysis (Table 1) were examined by WISH (A, Group I; B, Group II; C, Group III; D, Group IV). Embryos from crosses between *en‐pou5f3* ^+/−^ fish and wild‐type fish were subjected to *en‐pou5f3* induction at 90% epiboly (A, B) or 3‐ss (C, D), and after further development at 25°C for 1 h, the expression patterns of genes of interest were stained. After observations, respective embryos were genotyped. Dorsal views with anterior to the top. For each gene, the expression in wild‐type sibling (sib) and *en‐pou5f3* ^+/−^ (en‐pou) embryos are shown on the top and bottom, respectively. The numbers of embryos showing the indicated expression patterns and total scored embryos are shown in the bottom right. di, diencephalon; dmb, diencephalon‐midbrain boundary; fb, forebrain; mb, midbrain; nc, neural crest; np, neural plate; pnc, proneural cluster. Scale bar, 200 μm.

The reliability of the microarray data was further evaluated by WISH. Embryos from crosses of *en‐pou5f3* ^+/−^ and wild‐type fish were subjected to heat shock as described above, and the expression of the genes showing significant changes in the microarray was examined (Figure 3). Regarding the downregulated genes at 90% epiboly (Group I, *her3*, *pax6b*, *otx1a*, *hesx1*, and *foxb1a*), the characteristic expression in the neural plate was abrogated (Figure 3A), which is in keeping with the microarray data.

Among the Group II genes, increases in expression were previously observed for *gbx2* and *egr2b* (Inomata et al. 2020) (Maekawa et al. 2024). The expression of *sox9b*, *pou3f3a*, and *her4.1* was shown here to be markedly elevated by *en‐pou5f3* at 90% epiboly (Figure 3B), being again well consistent with the microarray data. Of note, the expression of *sox9b* and *her4.1*, which was rather weak in wild‐type siblings at the stage examined, was induced specifically in the regions where they were previously shown to be expressed at later stages in normal embryos (*sox9b*, neural crest (Hans, Liu, and Westerfield 2004); *her4.1*, proneural cluster (Takke et al. Takke et al. 1999)), suggesting that *pou5f3* suppression does not induce ectopic expression but rather promotes precocious expression.

With respect to the genes whose expression was significantly altered at 3‐ss (Table 1C,D), their expression was sometimes too low to be detected by WISH in our hands. We were thus able to examine the expression of three Group III genes (*her3*, *zic4*, *barhl2*) and one Group IV gene (*sebox*) by WISH (Figure 3C,D). At this stage, *en‐pou5f3* indeed abrogated *her3* expression (Hans, Scheer, et al. 2004) and significantly downregulated *zic4* (Elsen et al. 2008) and *barhl2* (Scholpp et al. 2007) in the neural plate, whereas *sebox* was extensively upregulated throughout the embryo, again supporting the microarray data.

Therefore, the reliability of the microarray data was confirmed both quantitatively and qualitatively in most cases. Considering the expected negative effect of *en‐pou5f3* overexpression, the expression of Group I/III genes is likely upregulated or maintained, whereas Group II/IV genes are downregulated by endogenous *pou5f3* at 90% epiboly/3‐ss.

### 3.3 GO Analysis of the Genes under Regulation by *en‐pou5f3*

GO analysis was conducted to characterize the genes that showed marked changes in expression levels. First, in terms of the GO‐Biological Process (BP) (Table 2A), independent of the induction stage and the manner of regulation (downregulation or upregulation), transcriptional regulation was the most prominent category (decreased at 90%, 128 genes; elevated at 90%, 76 genes; decreased at 3‐ss, 142 genes; elevated at 3‐ss, 39 genes), followed by embryonic morphogenesis (decreased at 90%, 39 genes; elevated at 90%, 21 genes; decreased at 3‐ss, 42 genes; elevated at 3‐ss, 14 genes). Meanwhile, some gene categories were found to be particularly prominent at specific stages or manners of regulation. First, Group I contained genes related to hindbrain development (23 genes), whereas Group II included many genes associated with Notch signaling (11 genes; representatives were *deltaB*, *deltaD*, *notch2*, *her4.1*). Group III was characterized by genes associated with RNA metabolism (109 genes) and cell motion (22 genes). Group IV included genes associated with macromolecular complexes (13 genes) and chromatin (7 genes；representative were *h1fx, h2afx, h1f0, histh1l*).

**TABLE 2:** Gene ontology analysis of the genes showing changes in expression levels by *en‐pou5f3* induction.

A. Gene ontology biological process (GO‐BP)						
Expression	90% epiboly			3‐somite stage		
	Term	Count	*p*	Term	Count	*p*
Decrease	Regulation of transcription	128	2.1 × 10^−20^	Regulation of transcription	142	1.3 × 10^−29^
	Embryonic morphogenesis	39	7.8 × 10^−11^	Embryonic morphogenesis	42	3.0 × 10^−13^
	Hindbrain development	23	8.8 × 10^−15^	RNA metabolism	109	2.7 × 10^−22^
				Cell motion	22	9.5 × 10^−5^
Increase	Regulation of transcription	76	1.9 × 10^−6^	Regulation of transcription	39	7.3 × 10^−2^
	Embryonic morphogenesis	21	8.4 × 10^−4^	Embryonic morphogenesis	14	9.2 × 10^−3^
	Notch signaling pathway	11	3.1 × 10^−7^	Macromolecular complex assembly	13	3.6 × 10^−4^
				Chromatin	7	9.1 × 10^−4^

B. Gene ontology cellular component (GO‐CC)						
	90% epiboly			3‐somite stage		
	Term	Count	*p*	Term	Count	*p*
Decrease	Nuclear lumen	22	6.1 × 10^−6^	Nuclear lumen	21	2.3 × 10^−5^
				Microtubule	9	8.0 × 10^−3^
Increase	Cytoskeleton	19	4.1 × 10^−2^	Chromatin	8	5.1 × 10^−4^
	Chromatin	8	8.9 × 10^−3^			

C. Gene ontology molecular function (GO‐MF)						
Expression	90% epiboly			3‐somite stage		
	Term	Count	*p*	Term	Count	*p*
Decrease	Transcription regulator activity	113	4.3 × 10^−23^	Transcription regulator activity	122	3.7 × 10^−28^
				Helicase activity	14	1.1 × 10^−3^
				ATPase activity	15	7.7 × 10^−2^
				RNA binding	19	9.8 × 10^−2^
Increase	Transcription regulator activity	64	3.8 × 10^−6^	Transcription regulator activity	34	7.3 × 10^−2^

*Note:* Representative categories based on DAVID Bioinformatics Resources 6.7 for respective induction stages and the manner of changes of expression. The significance of the association was examined by the Fisher's exact test.

Regarding the GO‐Cellular Component (CC) (Table 2B), genes downregulated at either 90% epiboly (Group I) or 3‐ss (Group III) included many genes associated with the nuclear lumen (22 genes and 21 genes, respectively). Genes upregulated at both stages (Group II and Group IV) included many chromatin‐related genes (8 genes in either case). Thus, GO‐BP and GO‐CC analyses suggested that many chromatin‐related genes were upregulated at both stages by *en‐pou5f3*, and these genes included various histone families, such as the H1/H5 family, H2A family, H2B family, and H3 family (Table 3). Meanwhile, many genes upregulated only at 90% epiboly were associated with the cytoskeleton (Group II, 19 genes; *kif1b, kif5aa, jak1* etc.), while many genes downregulated only at 3‐ss were associated with microtubules (Group III, 9 genes; *kif11, tuba8l2, tubb5* etc.).

**TABLE 3:** Upregulation of chromatin‐related genes by *en‐pou5f3* induction.^a^

Gene symbol	Family	Signal log ratio	
		90% epiboly	3‐ss
*h1fx*	H1/H5 family	0.9	1.3
*hist2h2l*	H2B family	0.6	0.5
*mid1ip1*	H2A family	0.5	−0.2
*wu:fe01e06*	H3 family	0.5	2.1
*wu:fe37d09*	0.6	0.4	1
*h2afy2*	H2A family	0.3	0
*h2afx*	H2A family	0.3	0.3
*hmgiy*	HMG protein (isoforms I and Y)	0.1	0.3
*wu:fe11b02*	H2B family	−0.2	1.5

^a^ Genes identified as upregulated at 90% epiboly and/or 3‐ss following *en‐pou5f3* induction, as determined by the Wilcoxon signed‐rank test (*p* < 0.0025), were clustered as chromatin/chromosome‐related based on GO‐CC analysis using DAVID Bioinformatics Resources 6.7.

Regarding the GO‐Molecular Function (MF) (Table 2C), all four gene groups included many genes associated with transcription regulator activity (I, 113; II, 64; III, 122; IV, 34). Of note, Group III was characterized by genes related to helicase (14 genes; *ddx3*, *ddx21* etc.), ATPase (15 genes; *asp5d*, *ttnb* etc.), and RNA binding (19 genes; *dkc1*, *ptbp1b* etc.).

Clustering analysis was carried out based on the results described above to grasp the overall gene functions (Table S14). This analysis is to create several groups by summarizing relevant items based on the results from each GO analysis. As a result, it was confirmed that Groups I–III contained many genes involved in transcriptional regulation and developmental control (I, transcriptional regulation, developmental regulation, cell motility; II, transcriptional regulation, developmental regulation, chromatin; III, transcriptional regulation, developmental regulation, neurogenesis). In contrast, Group IV was characterized by genes associated with chromatin, metal response, and translational control.

To know the signaling pathways triggered by *pou5f3*, pathway analysis was performed (Table 4). Regarding the genes regulated at 90% epiboly, Group I included genes involved in the TGF‐β and Hedgehog pathways, while Group II was enriched with genes involved in the MAPK pathway, lysosome pathway, and the Notch pathway. The enrichment of Notch pathway genes is consistent with the results of the GO‐BP analysis. In terms of the genes regulated at 3‐ss, Group III was characterized by the genes related to RNA polymerase and meiosis, whereas Group IV included genes involved in the MAPK, Toll, and ErbB signaling pathways.

**TABLE 4:** Pathway analysis of the genes whose expression was significantly altered by *en‐pou5f3* induction in embryos.^a^

Expression	90% epiboly			3‐somite stage		
	Term	Count	*p*	Term	Count	*p*
Decrease	TGF‐β	9	1.8 × 10^−2^	RNA polymerase	5	8.9 × 10^−3^
	Hedgehog	6	6.7 × 10^−2^	Oocyte meiosis	12	3.5 × 10^−3^
				Cell meiosis	10	3.2 × 10^−2^
Increase	MAPK	20	1.2 × 10^−2^	MAPK	17	9.9 × 10^−4^
	Lysosome	14	8.5 × 10^−4^	Toll	6	7.7 × 10^−2^
	Notch	6	5.8 × 10^−2^	ErbB	6	9.1 × 10^−2^

^a^ The gene sets obtained by the microarray were subjected to the pathway analyses using DAVID Bioinformatics Resources 6.7. Major signaling pathways are shown for respective *en‐pou5f3* induction stages and the manner of expression changes. The significance of the association was examined by the Fisher's exact test.

Taken together, our microarray analysis showed that numerous genes are under positive or negative regulation by *pou5f3* at 90% epiboly and/or 3‐ss, and that these genes are involved in developmental control, transcriptional regulation, proliferation, neurogenesis, signal transduction, and chromatin organization.

### 3.4 Regulation of *her* Genes in *en‐pou5f3*‐Induced Embryos

InterPro analysis (Table 5), which identifies characteristic domain structures in the genes affected by *en‐pou5f3* induction, showed that many downstream genes encode transcription factors containing homeodomains, bHLH domains, zinc finger domains, and HMG domains. Of note, the bHLH proteins were predominantly of the Orange‐type (bHLH‐O), including Her/Hes family members, which generally suppress neuronal differentiation.

**TABLE 5:** InterPro analysis of the gene sets showing significant alterations in expression levels due to *en‐pou5f3* induction.^a^

Expression	90% epiboly			3‐somite stage		
	Term	Count	*p*	Term	Count	*p*
Decrease	Homeobox	48	4.1 × 10^−17^	Homeobox	55	1.0 × 10^−22^
	Zinc finger	21	4.9 × 10^−2^	bHLH	24	6.9 × 10^−10^
	bHLH	18	1.1 × 10^−5^	HMG (*sox*)	6	4.1 × 10^−3^
	HMG (*sox*)	8	4.4 × 10^−3^	Orange	6	1.2 × 10^−3^
Increase	bHLH	16	3.2 × 10^−5^	Zinc finger	18	8.8 × 10^−2^
	Homeobox	16	6.9 × 10^−2^	SH2 motif	6	8.1 × 10^−2^
	Orange (*her*)	5	5.6 × 10^−3^			

^a^ The gene sets obtained by the microarray were subjected to the InterPro analyses using DAVID Bioinformatics Resources 6.7. Major gene families are shown for respective *en‐pou5f3* induction stages and the manner of expression changes. The significance of the association was examined by the Fisher's exact test.

Interestingly, the types of *her* genes affected by *en‐pou5f3*, as well as the direction of their regulation, varied depending on the stage (Figure 1E). In Tg embryos heat‐shocked at 90% epiboly, five *her* genes, including *her 4.1*, were significantly activated (*her2/Hes2* orthologue, *her4.1*, *her4.2/Hes5*, *hey1*, *hey2*) (Group II), although they were hardly affected at 3‐ss. On the other hand, two *her* genes were strikingly downregulated (*her3* and *her5*) at both 90% epiboly and 3‐ss. Similar regulation by *en‐pou5f3* at 90% epiboly was already found in our previous study for three *her* genes (*her3*, *her4.1*, *her5*) (Inomata et al. 2020). Not only among *her* genes, but also among all genes showing altered expression, *her3* showed the most striking downregulation at both stages examined (Tables 1 and [Link], [Link]). Among the six *her* genes activated by *en‐pou5f3* at 90% epiboly, *her2, her4.1*, and *her4.2* are supposed to be dependent on Notch signaling (Takke et al. 1999; Cheng et al. 2015), whereas the two *her* genes downregulated by *en‐pou5f3* (*her3*, *her5*) are Notch‐independent (Geling et al. 2004; Hans, Scheer, et al. 2004; Stigloher et al. 2008; Schmidt et al. 2013) (Figure 1E). These findings suggest that Notch‐dependent *her* genes are repressed, whereas Notch‐independent *her gene*s are activated by endogenous Pou5f3.

As *her* genes are among pivotal regulators of neural development, we further placed our focus on another transcription factor family, the *sox* family (Table 6). Indeed, HMG proteins listed in the InterPro analysis were Sox proteins. Consistent with the results of this analysis, the data extracted from the microarray results revealed that *soxB1* genes (*sox2* and *sox3*), which are key neural specifiers, as well as other *sox* genes also regulating neural development (*sox11a/sox11b*, *sox21a/sox21b*) (Rimini et al. 1999), were downregulated by *en‐pou5f3* at both induction stages, with the only one exception being *sox1a*, which showed moderate upregulation. Similar effects of *en‐pou5f3* on *soxB1* expression were already observed in our previous study (Inomata et al. 2020). Interestingly, among the *soxE* genes, generally involved in neural crest development and cartilage formation (Chiang et al. 2001), *sox9a* was significantly downregulated, whereas *sox9b* was upregulated at 90% epiboly. In addition, the expression of *sox32* that promotes endodermal development (Lunde et al. 2004) was increased at 3‐ss. These findings suggest that Pou5f3 also regulates these *sox* subgroup genes, contributing to nonneural differentiation.

**TABLE 6:** sox genes significantly downregulated by *en‐pou5f3* induction.^a^

Gene symbol	Signal log ratio^b^		Group
	90% epiboly	3‐ss	
*sox1a*	1.7	1.5	II, IV
*sox2*	−3.1	−1.1	I, III
*sox3*	−1.9	−1.5	I, III
*sox9a*	−3.3	−0.9	I, III
*sox9b*	4.5	1.0	II, IV
*sox11a*	−1.5	−1.2	I, III
*sox11b*	−2.3	−1.6	I, III
*sox21a*	0.4	−1.3	III
*sox21b*	−1.7	−4.9	I, III
*sox32*	0.1	2.0	II, IV

^a^ Genes are listed when signal log ratios for alterations of mRNA levels due to *en‐pou5f3* induction were two‐fold or more (decrease or increase) at 90% epiboly and/or 3‐ss.

^b^ When there were different probes for given genes, the most significant values are chosen to avoid confusion.

### 3.5 Stage‐Specific Functions of *pou5f3* around the End of Gastrulation

We then aimed to uncover the stage‐specific functions of *pou5f3* around the end of gastrulation. Each gene group that was significantly downregulated or upregulated was subdivided into the following subgroups as described above: (a) genes commonly regulated at both 90% epiboly and 3‐ss (Tables [Link], [Link]), (b) genes uniquely regulated at 90% epiboly (Tables [Link], [Link]), and (c) genes uniquely regulated at 3‐ss (Tables [Link], [Link]). These subgroups were then analyzed using the functional annotation clustering with the revised DAVID algorithm (DAVID 6.8) (Table S15). This algorithm clusters genes based on their shared functional annotations derived from GO, Pathway, and InterPro analyses, facilitating the identification of biologically meaningful clusters and improving the interpretation of large gene datasets.

In terms of the genes downregulated at both stages, we found a variety of transcription factor genes, including homeobox genes, HLH protein genes, and *sox* genes. The HLH genes include *her3* and *her5*, while the *sox* genes include *sox2* and *sox3*. This finding is consistent with the above analysis of both Group I and II, particularly the InterPro analysis (Tables 5, 6). Meanwhile, genes downregulated specifically at 90% epiboly include *zic* genes encoding zinc finger transcription factors (*zic2b, zic2a, zic5*) and TGF‐β family genes (*bmp2a/2b* and *lefty1/2*), aligning with the analysis of Group I genes. In contrast, genes downregulated specifically at 3‐ss include *pax* genes, such as the MHB‐forming gene *pax2a*, as well as Wnt‐signaling genes (*zfd8a, wnt11, wnt7ba*) and genes involved in neural development. Notably, irrespective of the stage, transcriptional regulators were prominently regulated by *pou5f3*.

Regarding the upregulated genes, only a small number of genes (24 genes) were commonly regulated as stated above, and functional annotation clustering did not yield any categories. The genes specifically regulated at 90% epiboly and at 3‐ss were analyzed in the same manner. Among the genes upregulated specifically at 90% epiboly, *her/Hes* genes (bHLH‐Orange) formed the top‐ranked group, followed by homeobox genes and bZIP transcription factor genes. These results were similar to those of the InterPro analysis of Group III (Table 5). In contrast, the genes upregulated specifically at 3‐ss showed greater diversity. Notably, the enrichment scores were relatively low, indicating that the regulated genes were highly diverse. This is likely why the results did not strongly align with the above‐mentioned analysis. Notably, transcription factor genes were a minor component. Instead, they included those encoding histones, which constitute nucleosomes, as observed in the GO analysis for this stage (Tables 2, 3), as well as those encoding peptidase inhibitor and extracellular matrix proteins. These genes are likely involved not in developmental regulation or cell differentiation, but rather in the functions of differentiated cells.

Overall, although different tendencies were observed in some cases, likely because of separate analyses of subgroups from larger groups (particularly in the case of downregulated genes), restriction to significantly regulated genes, and differences in the algorithm and definition of gene categories, the above analysis identified commonly regulated genes, 90% epiboly‐specific genes, and 3‐ss‐specific genes downstream of *pou5f3*, reinforcing both the distinct and common functions of *pou5f3* before and after the end of gastrulation.

### 3.6 Roles of Pou5f3 in the Transcriptional Regulation of Developmental Regulatory Genes

**FIGURE 4:**
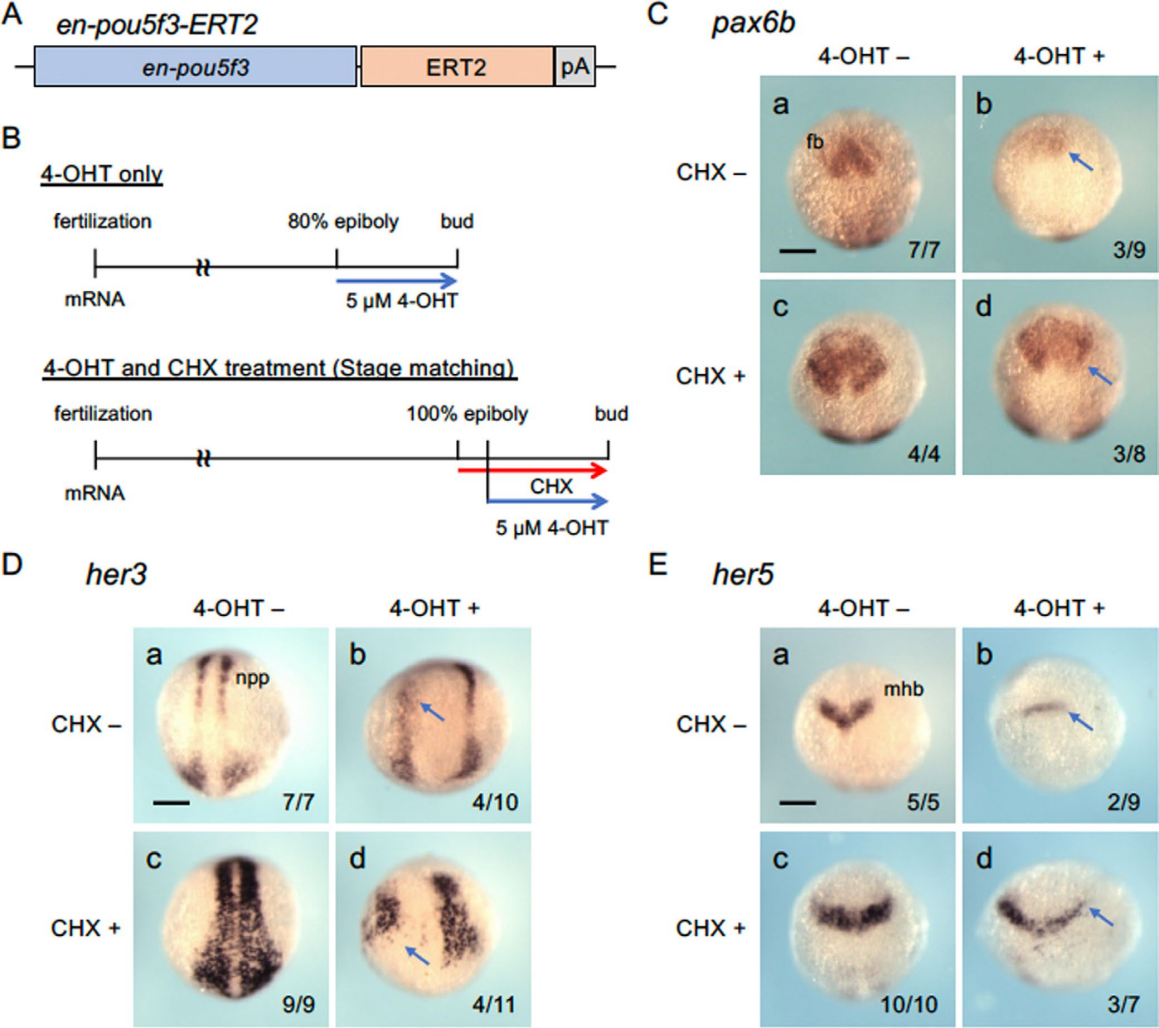
Regulation of downstream genes by activated En‐Pou5f3 in the absence of protein synthesis. A. Structure of En‐Pou5f3‐ERT2 overexpressed in embryos by mRNA injection. B. Time schedule of En‐Pou5f3‐ERT2 activation by 4‐hydroxytamoxifen (4‐OHT) and inhibition of protein synthesis by cycloheximide (CHX). Embryos injected with *en‐pou5f3‐ERT2* mRNA (150 pg/embryo) were treated for 2 h from 80% epiboly (8 hpf) with 4‐OHT, fixed at the bud stage, and examined for gene expression by WISH. To inhibit protein synthesis during activation, CHX was added 30 min before (7.5 hpf) addition of 4‐OHT (age matching). However, as development almost stopped as soon as CHX was added, CHX addition was delayed (100% epiboly) in some experiments (stage matching) so that the stage (bud stage) of staining was also at the bud stage. C–E. Expression of *pax6b* (C), *her3* (D), and *her5* (E) at the bud stage in embryos where En‐Pou5f3‐ERT2 was not activated (a, c) or activated (b, d) by 4‐OHT in the absence (a, b) or presence (c, d) of CHX. Dorsal views, with anterior to the top. Downregulation is marked with blue arrows. Numbers of embryos showing downregulation and total scored embryos are shown in the bottom right. fb, forebrain; mhb, midbrain‐hindbrain boundary; npp, neural progenitor pool; fb, forebrain. Scale bar, 200 μm.

We have shown that numerous genes are either activated or repressed by Pou5f3 in embryos around the end of epiboly. Several previous studies have suggested that Pou5f3 primarily acts as a transcriptional activator, both in embryos (Parvin et al. 2008; Khan, Nakamoto, Okamoto, et al. 2012; Onichtchouk et al. 2010) and in cultured cells (Kobayashi et al. 2018). To confirm the activator function of Pou5f3, we constructed a chimeric gene *en‐pou5f3‐ERT2* (Figure 4A), in which the *en‐pou5f3* sequence was fused to the sequence encoding a modified estrogen receptor, ER^T2^ (Feil et al. 1997). When embryos were injected with *en‐pou5f3‐ERT2* mRNA and treated with 4‐OHT from the sphere stage onward, the expression of *goosecoid* (*gsc*) in the dorsal shield (Stachel et al. 1993) expanded ventrally, whereas *eve1* expression in the ventral region (Joly et al., Joly et al. 1993) was reduced (Figure S1). As the induction of *en‐pou5f3* at the sphere stage dorsalizes embryos (Khan, Nakamoto, Okamoto, et al. 2012), we conclude that En‐pou5f3‐ERT2 is functionally activated by 4‐OHT.

Thus, we treated embryos expressing *en‐pou5f3‐ERT2* with or without 4‐OHT for 2 h and examined the expression of three Group I genes (*pax6b*, *her3*, *her5*) at the bud stage (Figure 4B). Although the effects were often asymmetric and mosaic, likely due to uneven distribution of the injected mRNA, all three genes were downregulated by 4‐OHT treatment, consistent with the results obtained in heat induction experiments (Figure 4C–E). When these embryos were pretreated with the protein synthesis inhibitor CHX, the expression levels of the markers were often higher than in untreated embryos. This increase is known as superinduction, a phenomenon considered to result from mRNA stabilization (Hershko et al. 2004). Notably, however, downregulation was still observed with 4‐OHT treatment. These results from *en‐pou5f3‐ERT2*/4‐OHT experiments further confirmed the activator function of Pou5f3.

To further assess the roles of Pou5f3, we examined whether major genes regulated by *en‐pou5f3* overlapped with those identified as Pou5f3‐bound targets by ChIP‐Seq analysis at pre‐MBT and/or post‐MBT stages (within 20 kb from the transcriptional start sites) (Table 1) (Leichsenring et al. 2013). We found that many of the downregulated genes contained Pou5f3 binding sites in their vicinity, whereas all of the upregulated genes examined lacked such binding sites.

### 3.7 The Upstream 4.0‐Kb DNA Is Sufficient for Regulating *her3* Expression in Embryos

**FIGURE 5:**
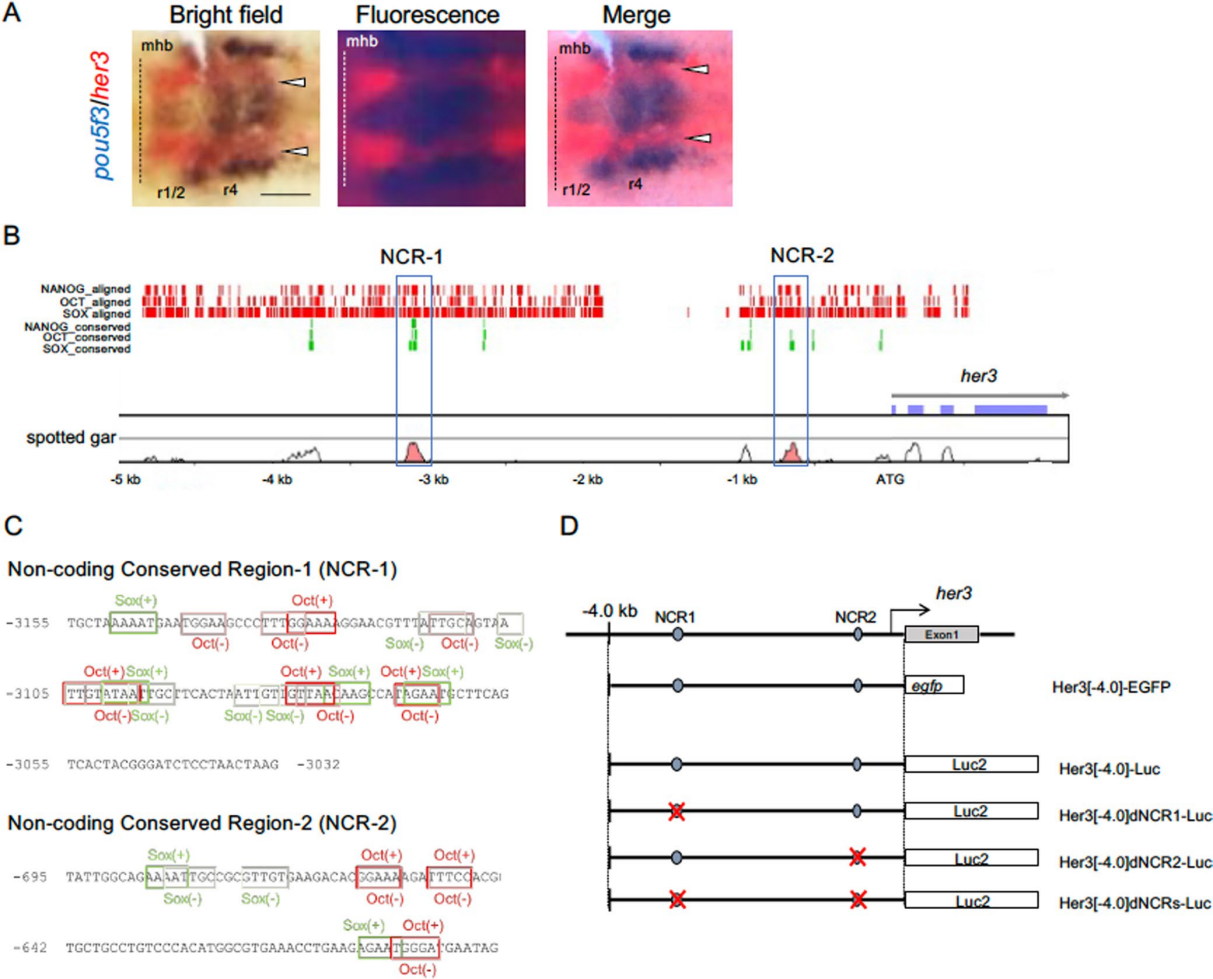
Noncoding conserved sequences found in the *her3* upstream DNA. A. Comparison of the expression patterns in the neural plate between *pou5f3* and *her3*. The expression of *her3*, stained red, was compared by two‐color WISH with that of *pou5f3*, stained in blue, at the bud stage. Dorsal views of flat‐mount preparations with anterior to the left. Broken lines mark the midbrain‐hindbrain boundary (mhb). Co‐expression of *pou5f3* and *her3* is marked with arrowheads. r1/2–4, rhombomeres 1/2–4. Scale bar, 100 μm. B. Comparison of the nucleotide sequences of the upstream 5.0‐kb DNA plus the coding regions between zebrafish *her3* and spotted gar *her3* using rVISTA. Prediction of the binding sequences for transcription factors (Oct, Sox, Nanog) were performed in parallel. Noncoding conserved regions (NCR‐1, NCR‐2) are marked with blue frames. Red vertical bars shown above indicate the zebrafish sequences to which transcription factors were predicted to bind, whereas green bars indicate the binding sites shared by the two species. C. Consensus transcription factor binding sites identified in the conserved sequences. Binding sites in the NCRs were searched by Match‐1.0 Public (vertebrates, cut‐off to minimize the sum of both error rates), and the sites for Oct (red) and Sox (green) are shown. ‘+’ and ‘–’ represent orientations of the binding sites. D. Structures of the constructs used in reporter assays. The genomic organization of *her3* is shown at the top. The two NCRs are shown with blue ovals. Below are shown the structures of the EGFP and luciferase reporter constructs, in which the upstream 4.0‐kb DNA of *her3* was ligated to the reporter genes. The EGFP reporter construct (Her3[−4.0]‐EGFP) was used for live imaging in embryos and cultured cells. The luciferase reporter Her3[−4.0]‐Luc and its deletion constructs, Her3[−4.0]dNCR1‐Luc, Her3[−4.0]dNCR2‐Luc, and Her3[−4.0]dNCRs‐Luc, were used in in vitro reporter assays.

We have suggested that *her3* is transcriptionally activated by Pou5f3 around the end of epiboly. *her3* is expressed in the hindbrain as two longitudinal stripes located between the medial and lateral proneural clusters in rhombomeres 1/2 and 4 (r1/2 and r4), which give rise to motoneuron and sensory neuron progenitors, respectively (Figure 5A) (Hans, Scheer, et al. 2004). In this study, we further confirmed by two‐color WISH that *pou5f3* is expressed broadly in the hindbrain, with relatively intense expression in proneural clusters (as reported previously) (Inomata et al. 2020) and weaker expression in the intervening region (arrowheads), where *pou5f3* is co‐expressed with *her3*.

To directly investigate how *her3* is regulated by Pou5f3, we compared the upstream genomic sequences of *her3* between zebrafish and spotted gar. A large‐scale comparison of the 10‐kb upstream DNA (Figure 5B) identified two highly conserved noncoding sequences from −3155 to −3032 bp (123 bp) and from −695 to −597 bp (98 bp) (relative to the ATG codon). These were designated as Noncoding Conserved Region 1 (NCR‐1) and NCR‐2, respectively (Figure 5C; Figure S2). Furthermore, we found multiple octamer sequences (putative Pou5f3 binding sites) (Parvin et al. 2008) and Sox binding sequences. Importantly, octamer sequences and Sox sites are closely associated in both NCRs. As Pou5f3 and SoxB1 cooperatively activate the transcription of *pou5f3* (Kobayashi et al. 2018), it is likely that they also positively regulate *her3* transcription. Previous studies have shown that the upstream 4.7‐kb DNA of *her3* can recapitulate its endogenous expression pattern in embryos (Hans, Scheer, et al. 2004). Although two POU/SOX sites were identified within this upstream DNA (Figure S2), they lie outside the NCRs identified here and their functions were not addressed (Okuda et al. 2010). Another study showed binding of Pou5f3 and Sox2 to the promoter region of *her3* (Figure S2), which was further implicated in *her3* expression both in embryos and cultured cells (Onichtchouk et al. 2010). However, these binding sites also fall outside the NCRs we identified.

**FIGURE 6:**
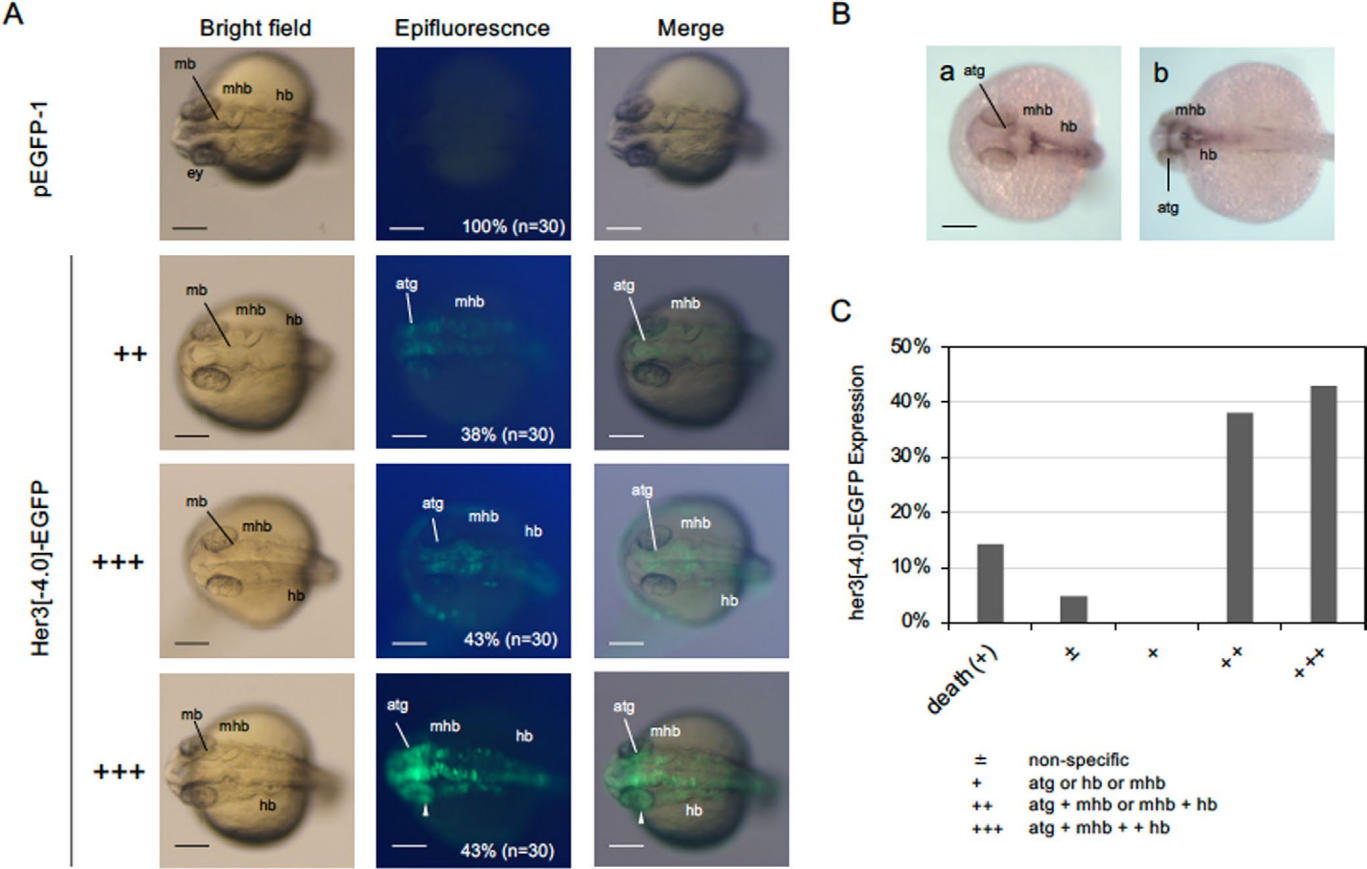
Expression of Her3[−4.0]‐EGFP in developing embryos. A. EGFP fluorescence in 24‐hpf embryos injected with pHer3[−4.0]‐EGFP DNA. Left column shows bright‐field images, the middle row shows fluorescence images, and the right column shows merged views. Dorsal views with anterior to the left. Expression rates and embryo numbers examined are shown at the bottom right. Scale bar, 200 μm. B. Endogenous expression of *her3* in the brain was examined by WISH at 24 hpf. Dorsal views of the anterior brain (a) and hindbrain (b) are shown with anterior to the left. C. Histogram showing fluorescence patterns in embryos injected with the reporter gene. The ordinate shows the percentages of embryos showing the expression patterns shown along the abscissa (*n* = 30). +, expression in the anterior tegmentum (atg), midbrain‐hindbrain boundary (mhb), or hindbrain (hb); ++, expression in atg and mhb or mhb and hb; +++, expression in atg, mhb, and hb. ±, nonspecific expression. mb, midbrain; ey, eye (optic vesicle). Two examples of +++ embryos and one example of ++ embryos are shown, whereas no embryos were scored as +. Weak ectopic expression observed when fluorescence was intense is marked with white arrowheads.

To assess the regulatory mechanism of *her3* and the role of *pou5f3*, we constructed an EGFP reporter gene in which the upstream 4.0‐kb DNA of *her3* was ligated to the *egfp* gene (Her3[−4.0]‐EGFP; Figure 5D). The expression of this reporter was examined in injected embryos during late somitogenesis (Figure 6A). We observed EGFP fluorescence specifically in the anterior tegmentum, MHB, and hindbrain (Figure 6A)—regions consistent with the endogenous expression pattern of *her3* as confirmed here by WISH (Figure 6B) and as previously reported (Hans, Scheer, et al. 2004). While weak ectopic expression was occasionally observed in adjacent regions, such as the eyes, particularly in embryos exhibiting strong EGFP fluorescence expression (+++), such expression is often unavoidable in transient reporter assays. Specific EGFP expression in the *her3* region was semiquantitatively scored as +++, ++, and +, resulting in 42.9%, 38.1%, and 4.8% of embryos, respectively (*n* = 30) (Figure 6C). These results indicate that the 4.0‐kb region—although slightly shorter than the 4.7‐kb region assessed in previous studies—is still sufficient to recapitulate the endogenous expression of *her3* in the brain primordium.

### 3.8 Transcriptional Regulation of *her3* Can Be Addressed Using Cultured Cells

**FIGURE 7:**
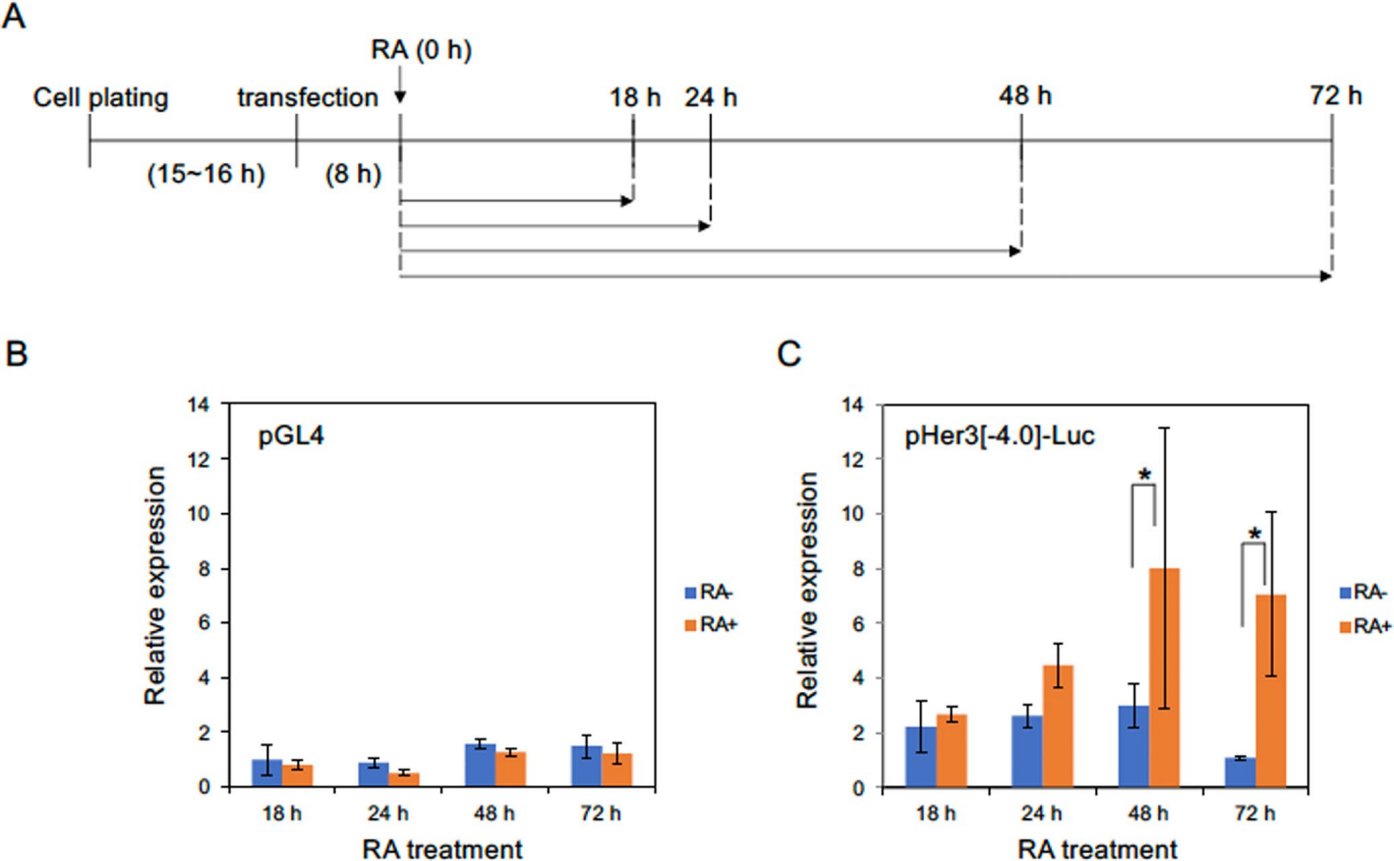
Transcriptional regulation of *her3* in P19 cells undergoing neural differentiation. A. Time schedule for inducing neuronal differentiation by exposure to retinoic acid (RA). Horizontal arrows indicate the durations after RA addition at 0 h. B, C. Expression of the backbone luciferase plasmid (pGL4, left) and pHer3[−4.0]‐Luc (right) after exposure of P19 cells to RA for the duration shown on the abscissa. The expression levels of firefly luciferase were standardized by *Renilla* luciferase expression as an internal control. Blue and orange bars indicate the reporter expression in the absence or presence of RA, respectively, relative to the expression of pGL4 at 18 h in the absence of RA. The experiments were repeated three times, resulting in essentially the same results. Error bars, standard deviations of means. *, *p* < 0.05.

While reporter assays in embryos provide valuable insights into the actual mechanisms of transcriptional regulation, their results can be influenced by numerous unknown factors and may be difficult to interpret. To circumvent this issue, we chose to analyze the transcriptional regulation of *her3* using a cultured cell system. A reporter construct was generated in which the 4.0‐kb upstream region was ligated to the luciferase (Luc) gene (Her3[−4.0]‐Luc; Figure 5D), and its expression was examined in mouse EC cells (P19 cells). These cells are capable of undergoing neuronal differentiation upon exposure to RA (Gao et al. 2001). To validate the use of this cell line for studying the regulatory mechanism of *her3* in developing neural precursors, RA was administered to the culture 8 h after transfection with the Luc reporter, and luciferase activity was examined over time (Figure 7A).

The backbone luciferase plasmid (pGL4) showed faint expression, regardless of the presence or absence of RA (Figure 7B). In contrast, the Her3[−4.0]‐Luc construct exhibited higher basal expression than the backbone construct, which remained relatively constant up to 48 h in culture but decreased by 72 h. Upon RA‐induced neuronal differentiation, Her3[−4.0]‐Luc expression gradually increased, peaking at 72 h, when the expression was significantly higher than that of control (Figure 7C). The RA‐dependent regulatory activity of the upstream 4.0 kb region in P19 cells was further confirmed using Her3[−4.0]‐EGFP (Figure S3). These results indicate that the 4.0‐kb DNA is sufficient to mediate transcriptional activation during neural differentiation, and that this in vitro system likely recapitulates the *her3* regulation in the developing neural tissues.

### 3.9 Transcriptional Regulation of *her3* by Pou5f3 and Sox3

As described above, *her3* appears to be positively regulated by Pou5f3, which was shown to function cooperatively with SoxB1 (Kobayashi et al. 2018). In this previous study, we demonstrated a physical interaction between Pou5f3 and Sox3 on the *pou5f3* enhancer. Furthermore, the expression of *sox2* and *sox3* is complementary and together encompasses the entire neural plate around the bud stage (Inomata et al. 2020). These findings prompted us to examine the roles of Pou5f3 and SoxB1 in regulating *her3* transcription in cultured cells (HEK293T cells, P19 cells). Notably, both cell lines have previously been used to successfully evaluate the transcriptional regulation of zebrafish genes (Kobayashi et al. 2018; Nakayama et al. 2017).

**FIGURE 8:**
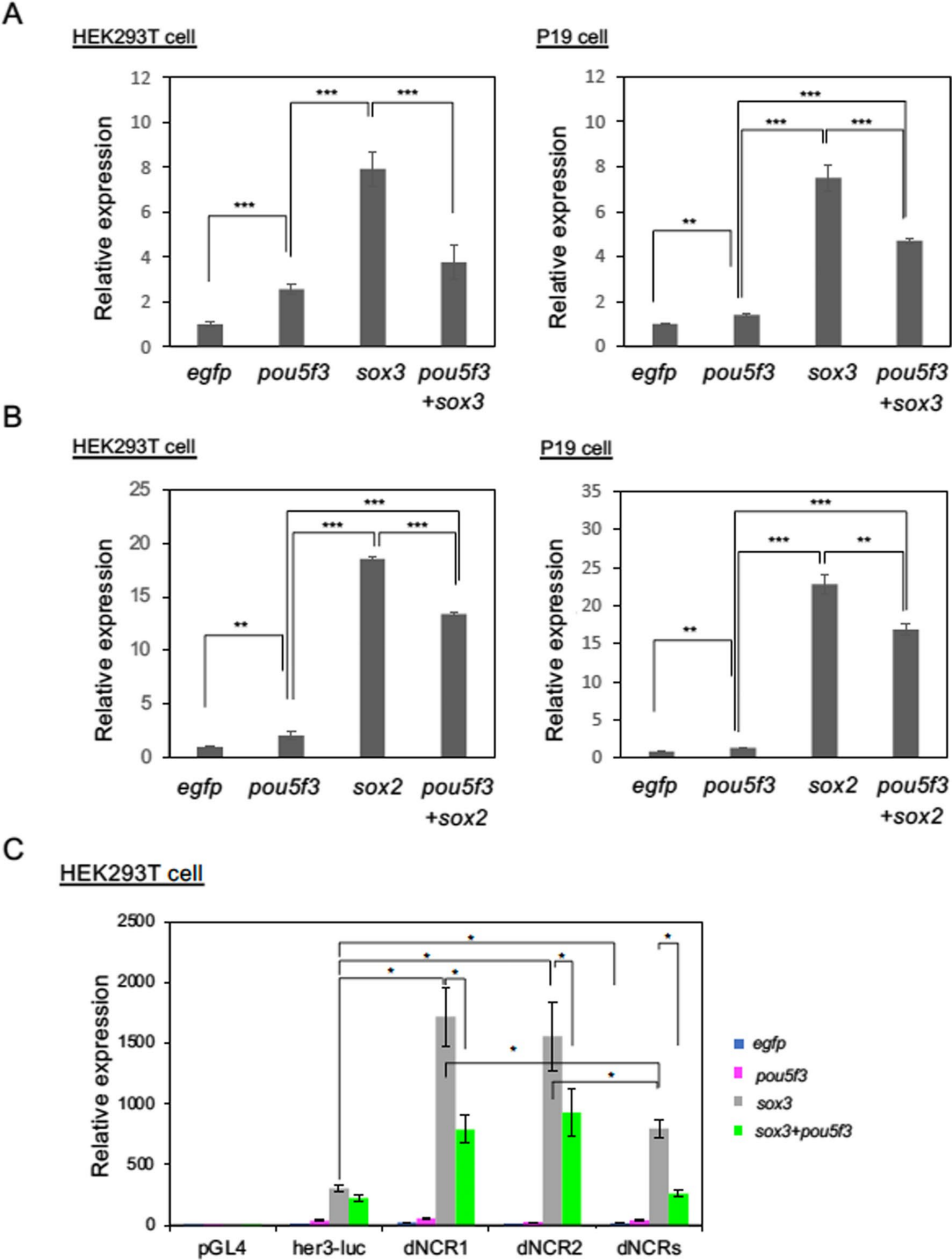
Transcriptional regulation of *her3* by Pou5f3 and SoxB1 in cultured cells. A, B. Effects of Pou5f3 and Sox3 (A) or Pou5f3 and Sox2 (B) on the expression of pHer3[−4. 0]‐Luc in HEK293T (left) or P19 (right) cells. The expression levels of the reporter in the presence of the effectors indicated on the abscissa are shown relative to the control level when *egfp* was used as an effector. C. Effects of Pou5f3 and Sox3 on the expression of luciferase constructs lacking NCR1, NCR2, or both. The expression levels of the reporter genes in HEK293T cells in the presence of the expression plasmids for *egfp* (blue), *pou5f3* (magenta), *sox3* (gray), and *pou5f3* plus *sox3* (green) are shown relative to the luciferase expression for pGL4 in the presence of *egfp*. The expression levels of firefly luciferase were standardized by *Renilla* luciferase expression as an internal control. The experiments were repeated three times, resulting in essentially the same results. Error bars, standard deviations of means. *, *p* < 0.05; **, *p* < 0.01; ***, *p* < 0.001.

First, we co‐transfected the Her3[−4.0]‐Luc construct with expression plasmids for Pou5f3 and/or Sox3 into HEK293T cells, observing 2‐fold activation by Pou5f3 and 8‐fold by Sox3 (Figure 8A, left). Interestingly, in contrast to the known synergistic effects between SoxB1 and Oct4/Pou5f3 in transcriptional regulation, co‐expression of Pou5f3 with Sox3 attenuated Sox3‐mediated activation to approximately 4‐fold. Similar results were obtained using P19 cells (Figure 8A, right), as well as with *sox2* (Figure 8B). The modest attenuation of Sox3‐dependent activation by Pou5f3 was further confirmed at the transcriptional level by qRT‐PCR analysis of luciferase mRNA in HEK293T cells (Figure S4). Together, these results suggest that Pou5f3 modestly activates *her3*, while SoxB1 factors are potent activators. Furthermore, Pou5f3 appears to mildly antagonize SoxB1‐mediated activation, suggesting a context‐dependent modulation of *her3* expression by Pou5f3 during neural development.

To investigate the functional significance of the two NCRs, we examined the expression of Her3[−4.0]‐Luc constructs lacking either or both NCRs in HEK293T cells (Figures 5D, 8C). In the absence of effector expression, all deletion constructs exhibited basal activity comparable with that of the full‐length construct. When Pou5f3 was co‐expressed, only weak activation was observed, similar to the intact construct. Interestingly, in the single‐deletion constructs lacking NCR‐1 or NCR‐2 individually (Her3[−4.0]dNCR1/2‐Luc), Sox3‐induced activation was markedly enhanced compared with the intact reporter, and this elevated expression was again suppressed by co‐expression of Pou5f3, reducing the activity to approximately half. The double‐deletion construct (Her3[−4.0]dNCRs‐Luc) showed a similar pattern; however, the Sox3‐mediated activation was less pronounced than in the single‐deletion constructs. Notably, the repression of Sox3‐induced activity by Pou5f3 in this context was even more prominent. The implications of these distinct responses of the luciferase constructs to Pou5f3 and Sox3 will be discussed below.

## 4 Discussion

### 4.1 Regulation of Zebrafish Development by the PouV Transcription Factor Pou5f3

Class V POU transcription factors have been implicated in diverse aspects of vertebrate development, particularly in cell differentiation or cell fate decisions (Onichtchouk 2016). In zebrafish, *pou5f3* is essential for isthmus development through regulation of the MHB‐forming gene network (Belting et al. 2001; Burgess et al. 2002). Subsequently, conditional suppression of the endogenous gene by *en‐pou5f3* induction has been used to dissect the stage‐specific functions of *pou5f3* during development (Khan, Nakamoto, Tai, et al. 2012). Of note, *en‐pou5f3* induction around the end of epiboly resulted in defects in the isthmic region. Importantly, the effects differed between late epiboly and early somite stages. We further confirmed that the molecular responses of the MHB region to *en‐pou5f3* induction change significantly between 90% epiboly and 3‐ss (Maekawa et al. 2024). At both stages, *en‐pou5f3* rapidly abrogated the expression of pivotal MHB‐specifying genes, including *pax2a*, *fgf8a*, and *en2a* (Nakamura 2001) (Rhinn and Brand 2001). When *en‐pou5f3* was induced at 90% epiboly, the expression of these genes was never restored at later stages. In contrast, induction at 3‐ss also led to initial downregulation, but their expression was subsequently restored.

These differences in expression recovery suggest a qualitative change in the chromatin states surrounding MHB‐forming genes, affecting their transcriptional regulation around the end of gastrulation. It is also possible that the effect of En‐Pou5f3 is not simple functional suppression of its endogenous counterpart, Pou5f3; the effects may be different between the two stages. However, we favor a view that En‐Pou5f3 acts similarly at both stages. This is supported by the fact that its immediate repressive effects on MHB‐forming genes were indistinguishable between 90% epiboly and 3‐ss.

### 4.2 Transcriptome Analysis of the Alteration in Gene Expression Elicited by Pou5f3 Suppression

The potential drastic changes of the regulatory role of *pou5f3* around the end of epiboly prompted us to comprehensively analyze the difference in the transcriptome change due to *pou5f3* suppression between 90% epiboly and 3‐ss. This analysis revealed numerous genes whose expression was significantly downregulated or upregulated at each developmental stage, which likely reflect the multifaceted roles of *pou5f3* during these developmental windows. Importantly, the data further confirmed at the molecular level that the function of *pou5f3* is strikingly different between late epiboly and early somitogenesis, as only limited fractions of genes showed consistent regulation across both stages.

Although microarray data are informative for understanding global gene networks in given biological aspects, it is always necessary to confirm the validity of the data, since such large‐scale analyses tend to sacrifice the preciseness of individual data. In this study, we reexamined the changes in expression levels/patterns for significantly affected genes using qRT‐PCR and WISH, which largely supported the microarray results and strengthened the reliability of our transcriptomic analysis. In addition, our current study quantitatively showed immediate downregulation of the MHB‐forming genes (signal log ratios at 90% epiboly and 3‐ss: *pax2a*, −2.6 and − 1.1; *fgf8a*, −2.4 and − 2.5; *en2a*, −2.2 and − 0.6, respectively) (Table S3), which well coincides with our previous observation (Maekawa et al. 2024).

*pou5f3* mRNA is maternally supplied and remains broadly expressed even in early gastrulae. Thereafter, its expression gradually becomes restricted to smaller domains, including proneural clusters within the neural plate, and eventually to the caudal‐most spinal cord (Yuikawa et al. 2021). Notably, despite this limited mRNA distribution, Pou5f3 protein remains broadly detectable by immunostaining even at early somite stages (Lippok et al. 2014). These broad and dynamic expression patterns likely underlie the diverse effects obtained following *en‐pou5f3* induction. Simple overexpression of wild‐type Pou5f3, by contrast, results in only mild phenotypic alteration (Takeda et al. 1994), including slight epiboly defects (Khan, Nakamoto, Okamoto, et al. 2012), suggesting that the function of Pou5f3 is not instructive but rather permissive (Burgess et al. 2002; Hauptmann et al. 2002). In this sense, the use of a dominant‐interference gene proved useful for revealing the functional contribution of Pou5f3 during embryogenesis.

Of note, previous microarray analyses identified a large number of potential *pou5f3* downstream genes in early embryos up to the mid‐gastrula stage (Onichtchouk et al. 2010). Only a limited number of genes were similarly affected in both their study (8 hpf) and ours (90% epiboly and 3‐ss), which is probably due to differences in the developmental stages analyzed as well as in the nature of *pou5f3* perturbation (absence of Pou5f3 up to 8 hpf vs. temporal suppression of Pou5f3 around the bud stage). Still, several genes were commonly identified, guaranteeing the reliability of our data and suggesting that at least some aspects of *pou5f3*‐mediated gene regulation are shared from early gastrulation through early somite stages. It is likely that the shared target genes correspond to those whose expression is affected in *MZ‐spg* embryos specifically around 8 hpf. The possible interference by *en‐pou5f3* with the functions of other octamer binding proteins cannot be entirely excluded, although the shared features of the microarray data between *en‐pou5f3* induction and *MZ‐spg* mutants support the specificity of the dominant‐interference approach employed in this study.

### 4.3 Possible Functions of *pou5f3* around the End of Epiboly Predicted by the Microarray Data

As discussed above, our microarray data are largely reliable, and the genes identified are likely downstream genes of *pou5f3*, even though the regulation may be direct or indirect. The data revealed significant alteration in expression levels for many genes across a wide range of gene categories, likely reflecting the multifaceted roles of *pou5f3* in a variety of regulatory mechanisms. Another important finding was the stage‐specific regulation of numerous genes by *pou5f3* at 90% epiboly and 3‐ss. To explore the potential functions of *pou5f3* at these stages, we performed GO, Pathway, and InterPro analysis. The most striking outcome from the GO analysis was the enrichment of numerous transcriptional regulators among *pou5f3*‐dependent genes. As these factors constitute complex gene regulatory networks, *pou5f3* probably functions as a regulatory hub, at least in late gastrulae and early somite‐stage embryos. Of note, at both stages, Pou5f3 was found to regulate a broad array of developmental regulatory genes, including genes involved in brain regionalization, consistent with previous findings that *pou5f3* is involved in a variety of developmental processes, such as isthmus development.

Pathway analysis, along with GO analysis, implicated many of the genes upregulated at 90% epiboly (Group II) in Notch signaling, which is involved in neurogenesis and other developmental processes (Beatus and Lendahl 1998; Weinmaster 1997; Louvi and Artavanis‐Tsakonas 2006). Furthermore, InterPro analysis revealed that the expression of several *her* genes, whether Notch‐dependent or ‐independent, is regulated by Pou5f3. These findings suggest that this *PouV* gene is likely involved in modulating Notch signaling and/or neural differentiation within the neural plate.

The above analyses of Groups I–IV thus revealed differences in the gene profiles induced by *en‐pou5f3* at 90% epiboly and 3‐ss, which is consistent with the potent distinct roles of *pou5f3* in MHB development. To further evaluate the molecular features of Pou5f3 targets specifically regulated at 90% epiboly and 3‐ss, each of the gene groups significantly downregulated or upregulated was subdivided into genes commonly regulated at both stages, those uniquely regulated at 90% epiboly, and those regulated at 3‐ss, which were then analyzed using the functional annotation clustering based on their shared functional annotations derived from GO, Pathway, and InterPro analyses.

Overall, although different tendencies were observed in some cases compared with the above‐mentioned analyses, this analysis identified commonly regulated genes, 90% epiboly‐specific genes, and 3‐ss‐specific genes downstream of *pou5f3*, further revealing both the distinct and common functions of *pou5f3* before and after the end of gastrulation. Although the mechanism is to be defined, this transcriptome shift likely underlies the different effects of Pou5f3 suppression before and after the end of epiboly. Elucidation of the process and mechanism of this regulatory shift will contribute to understanding the critical stage of MHB development, the end of epiboly, in vertebrate development.

It is worth noting that functional suppression of Pou5f3 appears to induce precocious expression of genes that are normally active at later stages of development, as observed for *sox9b* and *her4*. This finding suggests that one of the functions of Pou5f3 may be to repress these genes until the stages when they become required. This mechanism could also explain the upregulation of other genes such as *pth2r*, which are typically functional at later stages, although the possibility that they may have unknown earlier functions cannot be excluded.

### 4.4 Major Gene Groups Regulated by *pou5f3* around the End of Epiboly

Of note, some *her/Hes* genes act as core components of the Notch signaling pathway, whereas others suppress neural differentiation independently of Notch (Katoh and Katoh 2007). Alternatively, a single Her/Hes factor, such as mouse Hes1, functions in both Notch‐dependent and ‐independent manners depending on the cellular context (Baek et al. 2006; Stigloher et al. 2008). In zebrafish, *her4.1* inhibits neuronal differentiation in a Notch‐dependent manner (Stigloher et al. 2008). Indeed, it is expressed in proneural clusters and forms a feedback loop with *deltaD*, *deltaA*, and *notch1a*, thereby regulating neurogenesis through the control of proneural genes such as *neurog1* (Takke et al. 1999). For somitogenesis, *her1* and *her7* (*Hes7* co‐orthologues) function through Notch signaling (Holley et al. 2002; Oates and Ho 2002). In contrast, the functions of *her3*, *her5/her11*, *her6*, and *her9* are independent of Notch signaling. These genes are expressed in regions termed neural progenitor pools, which surround proneural cluster domains, and help maintain progenitor cells in an undifferentiated state (Bae et al. 2005; Geling et al. 2004; Hans, Scheer, et al. 2004; Ninkovic et al. 2005; Ohyanagi et al. 2025; Tsuruoka et al. 2025). Particularly, *her5* and *her11* are considered responsible for the immature state of the isthmus (Geling et al. 2004). Interestingly, *her3* has been identified as a direct target of *pou5f3* independently by two separate groups (Okuda et al. 2010; Onichtchouk et al. 2010).

The current analysis has demonstrated regarding *her* genes that *en‐pou5f3* induction at 90% epiboly upregulated *her2*, *her4.1*, *her4.2*, *hey1*, and *hey2* (Group II), suggesting their downregulation by endogenous Pou5f3 in late gastrulae. Of note, these *her* genes were not affected by *en‐pou5f3* at 3‐ss (Figure 1E), and thus their expression is refractory to Pou5f3 at the early somite stage. Interestingly, *her2*, *her4.1*, and *her4.2*, considered Notch‐dependent, as well as other Notch signaling‐related genes also belong to Group II (Table 2A), raising the possibility that Pou5f3 positively regulates neurogenesis through suppression of lateral inhibition in late gastrulae, which is consistent with the functions of *pou5f3* we previously revealed (Inomata et al. 2020). The striking difference in the competence of the neural plate to *pou5f3* regulation before and after the end of epiboly may at least partially underlie the transition regarding *pou5f3*‐mediated regulation of MHB development.

Meanwhile, *en‐pou5f3* induction at both 90% epiboly and 3‐ss significantly decreased the expression of *her3* and *her5*, making it likely that these Notch‐independent *her* genes are upregulated or maintained by Pou5f3 independent of the cessation of epiboly. As described above, Notch‐independent *her* genes contribute to the maintenance of neural progenitors and confine the proneural clusters, patterning neurogenesis in the neural plate, thus referred to as prepattern genes (Bae et al. 2005; Geling et al. 2004; Fisher and Caudy 1998; Stigloher et al. 2008; Ohyanagi et al. 2025; Tsuruoka et al. 2025). The *pou5f3* expression pattern in the neural plate is reminiscent of the proneural cluster patterns, but the *pou5f3* domain is larger and includes the surrounding neural progenitor pools (Inomata et al. 2020); thus, this gene may contribute to the maintenance of the progenitor pools through positively regulating Notch‐independent *her* genes.

In the current study, the most significantly downregulated gene was *her3*, the zebrafish orthologue of mouse *Hes3*. Such strong dependency of this gene on *pou5f3* was also reported for blastulae and pre‐gastrulae in the previous transcriptome analysis using *MZ‐spg* (Onichtchouk et al. 2010). These observations are compatible with the *her3* expression in the progenitor pools where *pou5f3* is weakly expressed (Inomata et al. 2020). Interestingly, mouse *Hes3* and human *HES3* are regarded as the *Oct4*‐downstream gene in ESCs (Onichtchouk et al. 2010; Boyer et al. 2005). The dependence of the expression of these *her/Hes* genes on *PouV* genes, shared among mammals and zebrafish, implies the common pivotal roles of *Hes3/HES3/her3* in the gene regulatory network involving *PouV* genes.

Recently, *her3* mutants were shown to survive to fertile adults (Kent et al. 2023). They were smaller with defects in the eyes at 24 hpf; still, they showed recovery by 72 hpf. This is surprising because both our study and the previous work showed that *her3* is the main target of *pou5f3* during early development. Actually, there are multiple Notch‐independent *her* genes in zebrafish, which were not severely affected or even slightly upregulated by *en‐pou5f3* induction (Figure 1E), and they may compensate for the lack of *her3*. Indeed, we recently generated *her5* mutants, which could grow to fertile adults like *her3* mutants (Ohyanagi et al. 2025). The possibility of functional compensation can be addressed using compound *her* mutants.

We also found that a variety of *sox* genes were significantly downregulated at both 90% epiboly and 3‐ss, suggesting their dependence on *pou5f3* in embryos. Among these are two *soxB1* genes (*sox2* and *sox3*), reinforcing the idea that *pou5f3* functions in the positive regulation of neurogenesis (Inomata et al. 2020). Although there are as many as six *soxB1* genes in zebrafish, among which *sox2/3/19a/19b* were implicated in neural development (Okuda et al. 2010), the present study has suggested essential roles of *sox2* and *sox3* in *pou5f3*‐mediated regulation of neural development. Interestingly, *sox1a*, another zebrafish *soxB1* gene expressed in the neural plate (Okuda et al. 2006), was upregulated instead at 90% epiboly, suggesting its unique function in neural development among the *soxB1* genes.

*en‐pou5f3* also significantly affected the expression of *sox21b* and *sox11a/b*, which belong to different groups of *sox* genes (*soxB2* and *soxC*, respectively) but are considered to regulate neural development (Kamachi et al. 2000). Of note, these genes are expressed in the MHB region (Rimini et al. 1999), implicating them in *pou5f3* regulation of MHB development. Among the *sox* genes involved in the differentiation of neural crest/cartilages (Chiang et al. 2001), *sox9a* was significantly downregulated, whereas *sox9b* was upregulated at 90% epiboly. Such contradictory regulation of these two *soxE* genes is puzzling, which still suggests the involvement of *pou5f3* in neural crest development. In this regard, it should be mentioned that these two genes are expressed in distinct sites in zebrafish embryos. Possible regulation of *sox32* by *pou5f3* is also interesting since *pou5f3* is required for endodermal development (Khan, Nakamoto, Tai, et al. 2012; Lunde et al. 2004; Reim et al. 2004).

Possible Pou5f3‐regulated genes are also enriched with chromatin‐related genes, especially histone genes, which were marginally upregulated by *en‐pou5f3* at both two induction stages. This suggests their negative regulation by Pou5f3, which may result in chromatin opening. Indeed, Pou5f3 contributes to ZGA as a pioneer factor through collaboration with Sox19b and Nanog (Pálfy et al. 2020; Riesle et al. 2023) (Miao et al. 2022). Such epigenetic regulation could result in the transition of the MHB‐related gene regulation around the end of epiboly, which was suggested previously (Maekawa et al. 2024) and in the current study. It is also possible that Pou5f3 regulates cell proliferation through regulation of histone synthesis. Of note, pathway analysis suggested Pou5f3 regulation of the genes involved in many signaling pathways, such as TGF‐β, hedgehog, MAPK, Toll, and ErbB pathways. The potential involvement of *pou5f3* in epigenetics, cell proliferation, and signaling pathways is interesting considering the diverse functions of *pou5f3* in development, although its biological significance needs to be addressed further.

### 4.5 Direct Regulation of the Downstream Genes by *pou5f3*

In the current study, to know if the regulatory function of Pou5f3 is via direct transcriptional regulation or mediated by secondary factors, we examined the effects of activating En‐Pou5f3‐ERT2 by 4‐OHT in the absence or presence of CHX. The usefulness of this ERT2/4‐OHT system was confirmed by its effects on dorsoventral patterning in early embryos. Pou5f3‐ERT2 activation downregulated three Group I genes (*pax6b*, *her3*, and *her5*), again supporting the microarray data. We obtained essentially the same results even when En‐Pou5f3‐ERT2‐expressing embryos were treated with 4‐OHT in the presence of CHX, strongly suggesting that Pou5f3 directly regulates gene expression positively, even though the possibility of indirect regulation cannot be fully excluded. Indeed, Class V POU factors have been considered a transcriptional activators (Bakhmet and Tomilin 2022), which was supported for zebrafish Pou5f3 as well (Parvin et al. 2008; Onichtchouk et al. 2010; Khan, Nakamoto, Okamoto, et al. 2012; Kobayashi et al. 2018), making it highly likely that the activation of genes, such as Group II/IV, by *en‐pou5f3* results from indirect regulation. Unfortunately, however, similar studies using Pou5f3‐ERT2/CHX have been unsuccessful so far because the effects were unclear and require improvement of the experimental procedures. Direct regulation of Group I/III genes, not of Group II/IV genes, by Pou5f3 was further supported by the fact that Pou5f3 binds only to the upstream DNA of the former genes, as shown by the ChIP assay. Similar direct regulation was also shown previously using *MZ‐spg* embryos for the genes that were positively regulated by Pou5f3 (Onichtchouk et al. 2010).

However, the roles of *pou5f3*, suggested by the microarray and *pou5f3‐ERT2*/4‐OHT experiment, may suffer from problems inherent in in vivo experiments. *en‐pou5f3*/*en‐pou5f3‐ERT2*/4‐OHT will also affect other genes, interfering with the direct effects of *en‐pou5f3* in embryos. In the case of microarray experiments, whole embryos were used, which could complicate the effects of *en‐pou5f3* induction. To further assess such transcriptional regulation by Pou5f3, we employed in vitro reporter assays. As stated above, the two cell lines employed here were already used previously to demonstrate the transcriptional regulation of zebrafish genes (Kobayashi et al. 2018; Nakayama et al. 2017). In addition, HEK293T cells are derivatives of HEK293 cells, which exhibit neuron‐like properties (Shaw et al. 2002), supporting their relevance for this analysis.

The upstream 4.7‐kb DNA of *her3* was previously shown to recapitulate *her3* expression in embryos (Hans, Scheer, et al. 2004). Here, the 4.0‐kb DNA upstream of *her3* was confirmed by in vivo EGFP reporter assay to conduct the same spatial regulation in the anterior tegmentum, MHB, and hindbrain. This 4.0‐kb DNA‐driven transcription was significantly enhanced in P19 cells during RA‐induced neural development, thus validating P19 cells as an excellent system for analyzing *her3* regulation in the neural progenitors in the neural plate and that the 4.0‐kb DNA harbors regulatory activity in the neural tissue.

In this study, we identified two regions conserved among teleost fish (NCRs), both of which included multiple Oct/Sox binding sites. The in vitro reporter assays using both HEK293T cells and P19 cells showed that *her3* was activated only marginally by *pou5f3* alone, but extensively in the presence of *sox3*, which is in line with the broad expression of *sox2* and/or *sox3* in the midbrain‐hindbrain region where *her3* is expressed (Inomata et al. 2020). Notably, *pou5f3* slightly weakens the *sox3*‐mediated activation of *her3*. These results are seemingly inconsistent with the microarray data and the effects of *en‐pou5f3‐ERT2*/4‐OHT (Figure 4D). However, despite this weak suppression of SoxB1‐mediated *her3* activation by Pou5f3, Her3[−4.0]‐Luc expression was still much higher than that caused by Pou5f3 alone, suggesting that the upregulation of *her3* by Pou5f3 in embryos, implied by the effects of *en‐pou5f3* induction, depends on the synergy between Pou5f3 and other factors, such as SoxB1. Strong enhancement of *her3* expression by SoxB1, suggested by the in vitro reporter assay, may explain the drastic En‐Pou5f3‐dependent downregulation of *her3* in embryos, shown by the microarray analysis, as *soxB1* was also downregulated by *en‐pou5f3* induction (Table 6). In addition, this Pou5f3‐mediated suppression of *her3* activation by SoxB1 may at least partially explain why *her3* is expressed in the progenitor pools where *soxB1* is expressed, but not in proneural clusters where *pou5f3* is strongly expressed in addition to *soxB1*. It is likely that the Pou5f3‐SoxB1 interaction is based on their physical interaction on the regulatory sequence. Indeed, we observed such interaction on the *pou5f3* enhancer (Kobayashi et al. 2018), but it needs to be examined in the future on the *her3* enhancer as well.

It has been well established that PouV and SoxB1 activate transcription cooperatively in mice (Ambrosetti et al. 2000; Kuroda et al. 2005; Rodda et al. 2005; Ng et al. 2012) and zebrafish (Kobayashi et al. 2018; Leichsenring et al. 2013; Lee et al. 2013). Cooperative regulation of *her3* by Pou5f3 and SoxB1 observed here, which was also reported before (Onichtchouk et al. 2010), is another example, but negative synergy between PouV and SoxB1 is a novel finding, to the best of our knowledge. There is a limitation when interpreting these results as the regulation of zebrafish genes was assessed in vitro using human cells. However, we observed typical synergy between Pou5f3 and SoxB1 in the regulation of the *pou5f3* promoter (Kobayashi et al. 2018), and the unexpected interaction between these factors on the *her3* promoter likely represents its inherent property. This intriguing finding may provide new insights into the roles of the cooperative action of PouV and SoxB1 in vertebrate development.

A previous study revealed two elements (distal and proximal) in the upstream of *her3* which include SOX‐POU composite sites, although their regulatory activities were not addressed (Okuda et al. 2010) (Figure S2). Another study identified a SOX‐POU element near the core promoter, which was shown in embryos and HEK293 cells to be necessary for a full regulatory activity (Onichtchouk et al. 2010) (Figure S2). Meanwhile, we identified two conserved sequences in the upstream DNA (NCRs), which did not coincide with the previously identified SOX‐POU sites, suggesting a multilayered regulatory mechanism of *her3* involving SOX/POU factors. It is possible that the previously identified *cis*‐elements and those we found here play distinct roles in embryos. However, they are closely located and interdigitated, as is shown in Figure S2, and likely regulated similarly by Pou5f3 and SoxB1, which are both broadly expressed in early embryos and across the neural plate. We therefore favor the view that these elements act redundantly and are repeatedly utilized during development, akin to shadow enhancers described in many developmental genes (Kvon et al. 2021). Nevertheless, their potential functional divergence remains an open question, best addressed through in vitro and in vivo reporter assays, including stable reporter expression analysis in Tg fish.

The functional significances of these NCRs were suggested by the expression of deletion constructs in HEK293T cells. Deletion of either or both NCRs did not affect the expression of the original construct (Her3[−4.0]‐Luc) in the absence of effectors. Marginal upregulation by Pou5f3 was not affected either. However, the same deletions significantly reinforced Sox3‐dependent activation of the reporter constructs. Thus, the NCRs are not essential for *her3* activation by Pou5f3 and/or Sox3 but may suppress the Sox3‐dependent enhancer activity of the 4.0‐kb region. However, as Sox3‐dependent activation of the double‐deleted construct was not so striking compared with that of the single‐deleted constructs, the two NCRs may also contribute to positive regulation by Sox3 besides negative regulation. The attenuation of the Sox3‐dependent activation by Pou5f3 was more striking in the case of the deleted constructs, particularly for the double‐deleted construct; NCRs may interfere with Pou5f3‐mediated suppression of the Sox3‐dependent *her3* upregulation. Thus, the functions of the NCRs we identified in this study seem important for integrating the regulation of *her3* transcription by SoxB1 and Pou5f3 together with the typical SOX‐POU composite sites identified previously.

### 4.6 Possible Conserved Roles of the PouV‐Her3/Hes3 Pathway in Vertebrate Neurogenesis

Zebrafish *her3* has been shown to maintain neural progenitor cells in the neural plate (Bae et al. 2005; Hans, Scheer, et al. 2004; Ohyanagi et al. 2025) and NSCs in the adult brain (Chapouton et al. 2011). Similarly, mouse *Hes3* contributes to the maintenance of early neural progenitors in the neural plate, particularly in the MHB/isthmus region (Kageyama et al. 2008), and supports the survival of neural precursors derived from the adult NSCs in response to either insulin or Dll4 (Androutsellis‐Theotokis et al. 2008). Notably, binding sites for POU5F1/Oct4 and SOX2/Sox2 have been identified in the promoters of human *HES3* and mouse *Hes3* (Katoh and Katoh 2007). Consistent with this, as stated in the Introduction, *HES3* and *Hes3* are regarded as downstream targets of *PouV* in ESCs (Onichtchouk et al. 2010; Boyer et al. 2005). These findings suggest that the PouV‐Hes3/Her3 pathway may play a conserved role in maintaining neural progenitors and NSCs across vertebrates.

## 5 Conclusion and Perspectives

In this study, we comprehensively analyzed *pou5f3* downstream genes in zebrafish embryos at 90% epiboly and 3‐ss. Our data identified numerous genes positively or negatively regulated by Pou5f3, with limited overlap between the two stages. Major components of the downstream genes encode developmental regulators, including those involved in brain regionalization and neuronal differentiation. Notably, we observed a striking stage‐specific difference in the competence of the neural plate to Pou5f3 suppression, demonstrating that the Pou5f3 function is temporally and dynamically regulated even within the short period around the end of epiboly. While the current data do not directly resolve the mechanism of this developmental switch, they offer important insights into the dynamic aspects of isthmus development—an area requiring further investigation. Understanding this mechanism may shed light on the context‐dependent pleiotropic functions of Class V POU factors in pluripotency maintenance, zygotic gene activation, later developmental regulation, and carcinogenesis (Patra 2020).

*her3* was already shown to be downstream of *pou5f3* up to mid‐gastrulation, but our study has further shown the dependence of this gene on Pou5f3 around the end of epiboly, implicating the *PouV‐Hes3/her3* pathway in various regulatory processes throughout early development. Pou5f3 regulation of *her3* was confirmed by in vitro reporter assays and 4‐OHT‐induced En‐Pou5f3 activation. Reporter assays further showed the activator function of Pou5f3, negative synergy between Pou5f3 and Sox3, and the significance of the two conserved sequences within the upstream DNA in the transcriptional regulation of *her3*.

The essential role of *Oct4/Pou5f1* in pluripotency is well known, although its involvement in brain formation is still elusive. We consider that our findings in this study will contribute to understanding *PouV*‐regulated brain development, especially MHB/isthmus development, not only in fish but also in mammals.

## Author Contributions

**Masaaki Ikeda:** conceptualization, data curation, formal analysis, investigation, methodology, visualization, writing – original draft. **Kana Kobayashi:** data curation, formal analysis, investigation, methodology, visualization. **Yukiko Nakayama‐Sadakiyo:** data curation, formal analysis, investigation, validation, visualization. **Yuto Sato:** investigation, validation. **Ayano Tobita:** investigation, validation. **Mika Saito:** investigation, validation. **Kyo Yamasu:** conceptualization, funding acquisition, project administration, resources; supervision, writing – review and editing.

## Conflicts of Interest

The authors declare no conflicts of interest.

## Supporting information


**Figure S1.** Functional inhibition of endogenous Pou5f3 in embryos by activation of En‐Pou5f3‐ERT2. Embryos injected with mRNA for *en‐pou5f3‐ERT2* or *egfp* mRNA (150 pg/embryo) were exposed to 4‐OHT according to the schedule shown in (A) and examined for dorsoventral patterning at the shield stage by the expression of *eve1* as a ventral marker and *gsc* as a dorsal marker (B). Bars show the dorsoventral extents of the expression of the markers. Lateral views with anterior to the top and dorsal to the right. The numbers of embryos showing indicated patterns and total numbers of scored embryos are shown at the bottom right. Scale bar, 200 μm.

**Figure S2.** The sequence of the upstream DNA of *pax2a* from −3974 to +3 bp is shown with positions relative to the ATG codon on the right. The positions −3000, −2000, and − 1000 are shown with red letters. The noncoding conserved sequences (NCR‐1 and NCR‐2) are shown in light blue. The Distal and Proximal SOX‐POU elements are marked with underlines and the primer sequences used for PCR amplification are shown in yellow (Okuda et al. 2010). The SOX‐Pou5f3 binding site and TATA box identified previously are shown in green and gray, respectively (Onichtchouk et al. 2010).

**Figure S3.** Fluorescence views of her3[−4.0]‐EGFP expression in P19 cells undergoing neuronal differentiation. P19 cells transfected with pGL4 or her3[−4.0]‐EGFP were plated onto 96‐well plates (5.6 × 10^3^ cells/well). After transfection, cells were cultured for 8 h and EGFP fluorescence was captured (A). Subsequently, cells were further cultured in the absence or presence of RA for 18 h and fluorescence was again detected (B). Cells with strong fluorescence were counted in five different frames and shown in the bottom left with standard errors.

**Figure S4.** Confirmation of the transcriptional regulation of *her3* by Pou5f3 and SoxB1 in cultured cells. Effects of Pou5f3 and SoxB1 on the expression of Her3[−4. 0]‐Luc in HEK293T cells were quantitated by qRT‐PCR. The mRNA levels of the luciferase gene in the presence of the expression plasmids for *egfp* (blue), *pou5f3* (magenta), *sox3* (gray), and *pou5f3* plus *sox3* (green) are shown relative to the luciferase expression in the presence of *egfp*. The mRNA levels of firefly luciferase were standardized by *Renilla* mRNA expression as an internal control. Error bars, standard deviations of means. *, *p* < 0.05; **, *p* < 0.01; ***, *p* < 0.001.

**Text S1.** (Methods).

Table S1.

Table S2.

Table S3.

Table S4.

Table S5.

Table S6.

Table S7.

Table S8.

Table S9.

Table S10.

Table S11.

Table S12.

Table S13.

Table S14.

Table S15.

## Data Availability

The data that support the findings of this study are available on request from the corresponding author. The data are not publicly available due to privacy or ethical restrictions.
