# Supplementary figures and images for: Transcriptome Analysis Suggested Striking Transition Around the End of Epiboly in the Gene Regulatory Network Downstream of the Oct4‐Type POU Gene in Zebrafish Embryos
Source: Dev Growth Differ. 2025 Jun 9;67(5):245–69. doi: 10.1111/dgd.70012 (PMC12199784; doi:10.1111/dgd.70012)

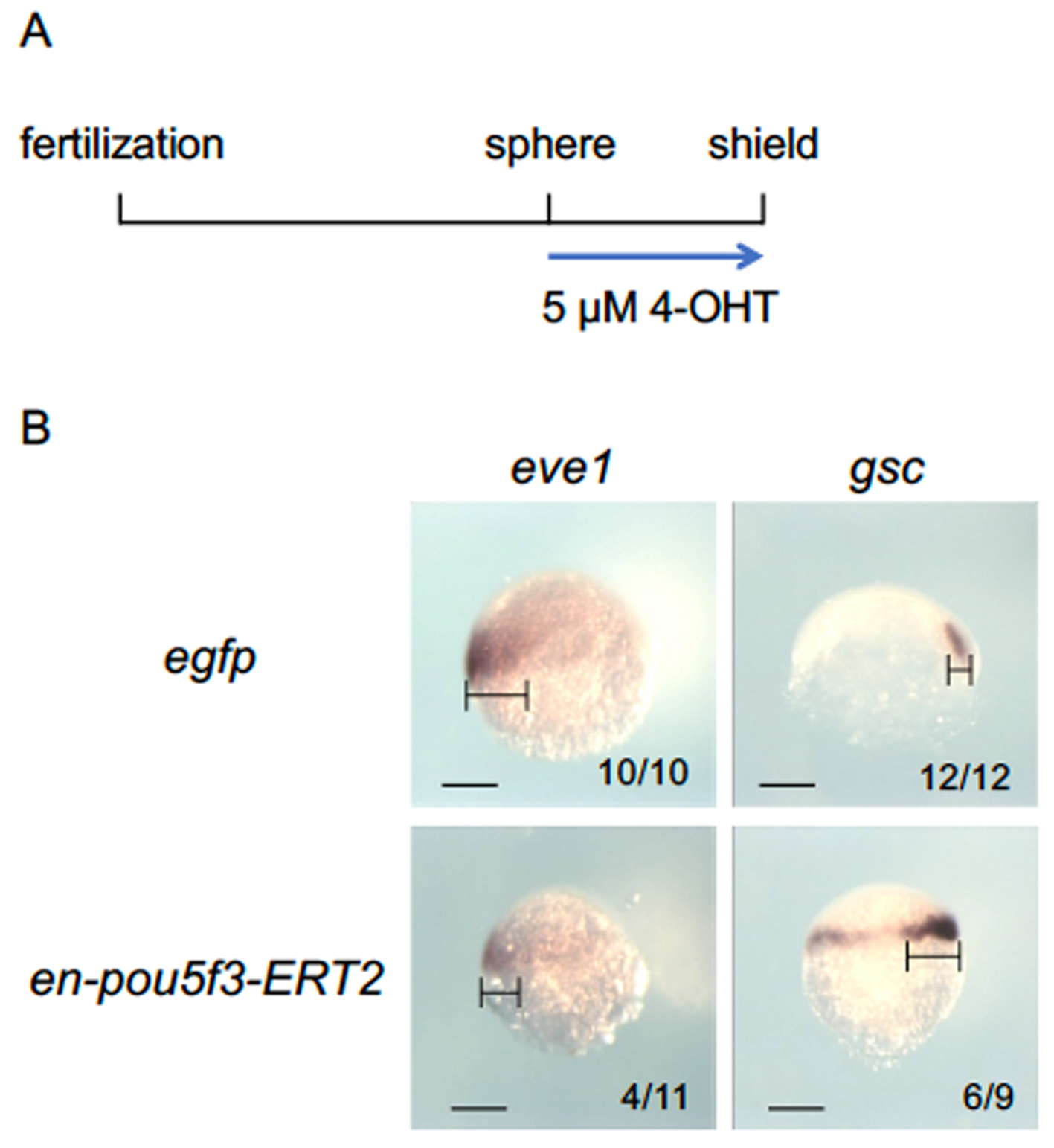

Supplement: Supplementary file 1 — Figure S1. Functional inhibition of endogenous Pou5f3 in embryos by activation of En‐Pou5f3‐ERT2. Embryos injected with mRNA for en‐pou5f3‐ERT2 or egfp mRNA (150 pg/embryo) were exposed to 4‐OHT according to the schedule shown in (A) and examined for dorsoventral patterning at the shield stage by the expression of eve1 as a ventral marker and gsc as a dorsal marker (B). Bars show the dorsoventral extents of the expression of the markers. Lateral views with anterior to the top and dorsal to the right. The numbers of embryos showing indicated patterns and total numbers of scored embryos are shown at the bottom right. Scale bar, 200 μm. [file DGD-67-245-s019.tif]

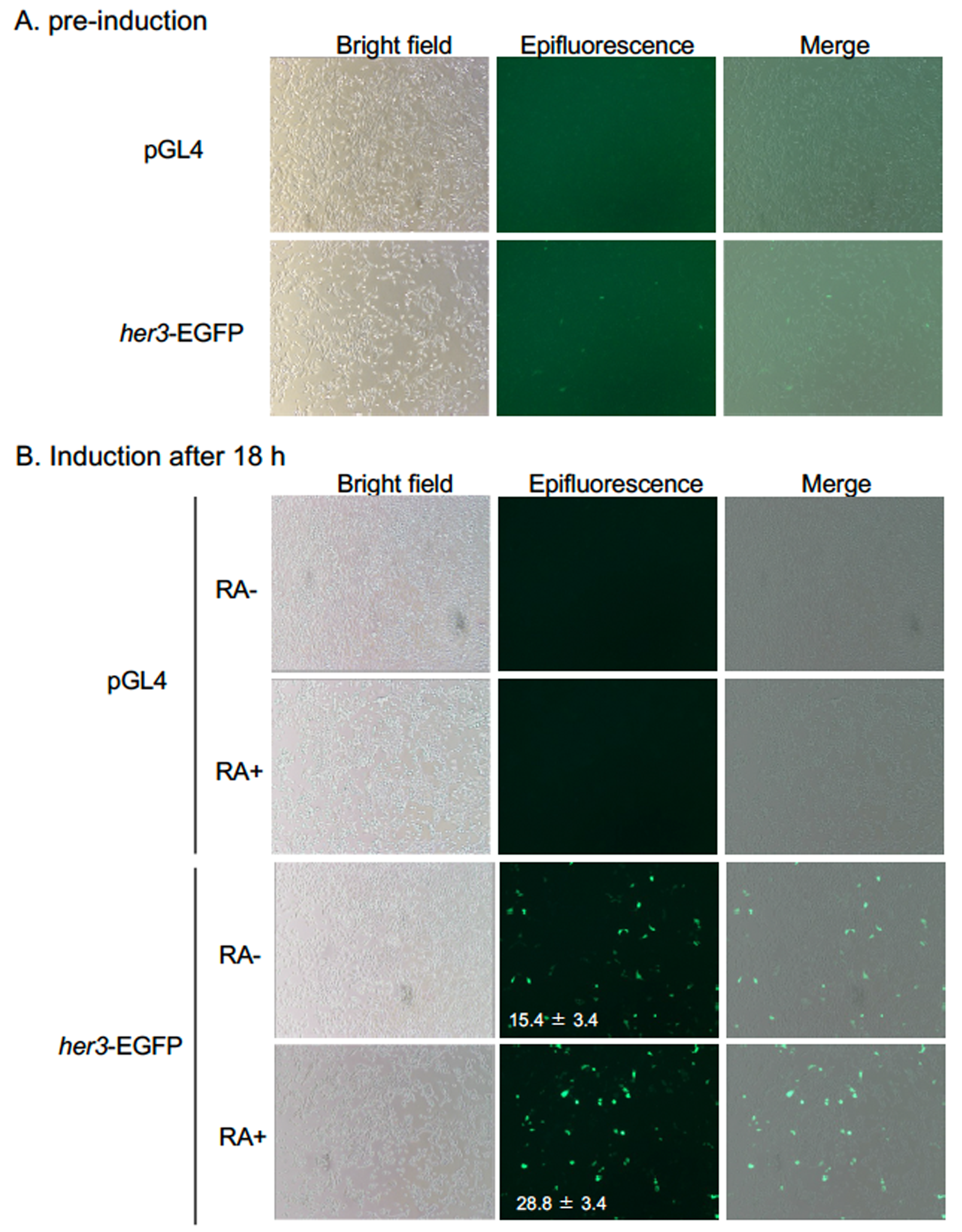

Supplement: Supplementary file 3 — Figure S3. Fluorescence views of her3[−4.0]‐EGFP expression in P19 cells undergoing neuronal differentiation. P19 cells transfected with pGL4 or her3[−4.0]‐EGFP were plated onto 96‐well plates (5.6 × 10^3^ cells/well). After transfection, cells were cultured for 8 h and EGFP fluorescence was captured (A). Subsequently, cells were further cultured in the absence or presence of RA for 18 h and fluorescence was again detected (B). Cells with strong fluorescence were counted in five different frames and shown in the bottom left with standard errors. [file DGD-67-245-s004.tif]

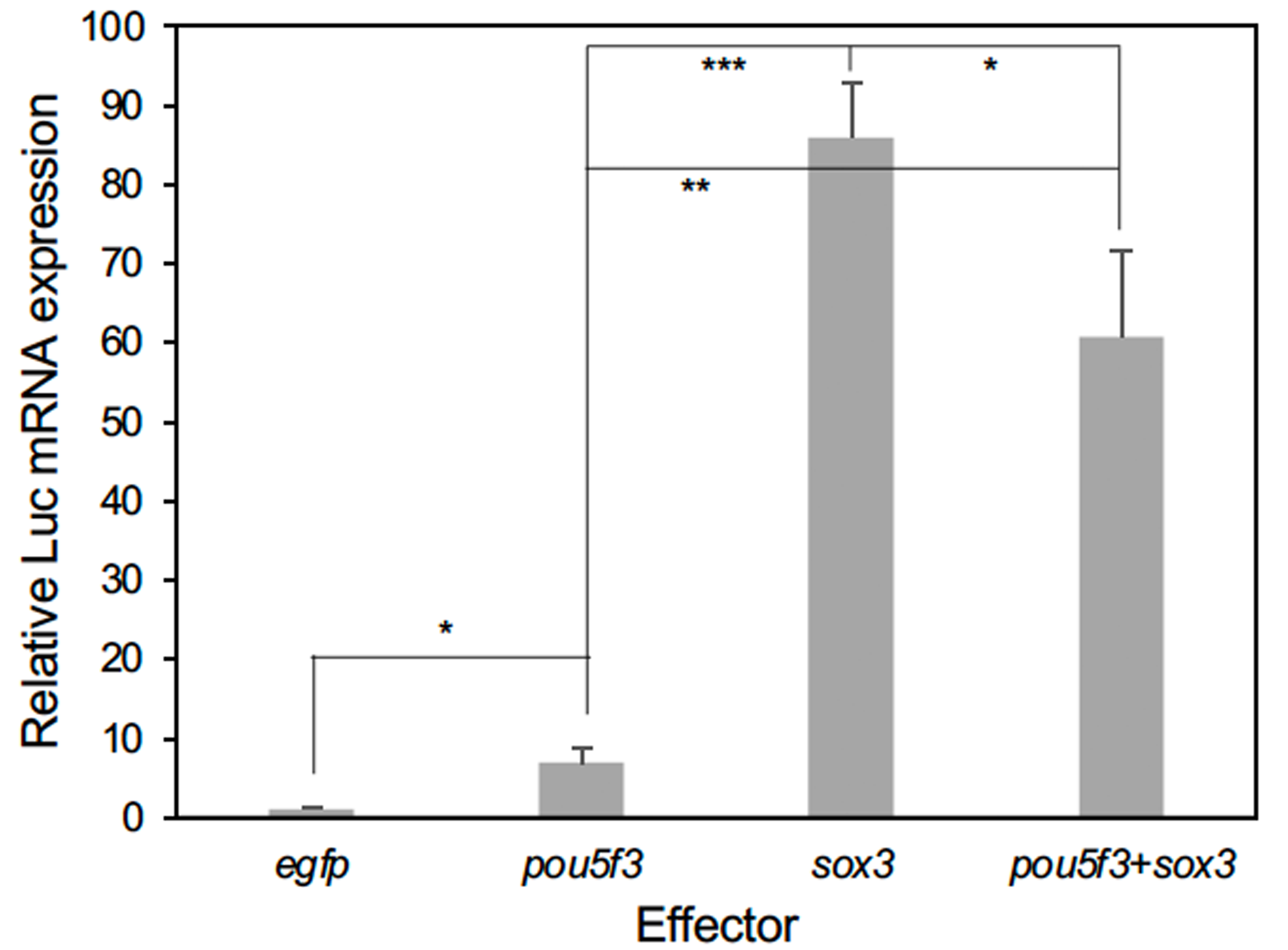

Supplement: Supplementary file 4 — Figure S4. Confirmation of the transcriptional regulation of her3 by Pou5f3 and SoxB1 in cultured cells. Effects of Pou5f3 and SoxB1 on the expression of Her3[−4. 0]‐Luc in HEK293T cells were quantitated by qRT‐PCR. The mRNA levels of the luciferase gene in the presence of the expression plasmids for egfp (blue), pou5f3 (magenta), sox3 (gray), and pou5f3 plus sox3 (green) are shown relative to the luciferase expression in the presence of egfp. The mRNA levels of firefly luciferase were standardized by Renilla mRNA expression as an internal control. Error bars, standard deviations of means. *, p < 0.05; **, p < 0.01; ***, p < 0.001. [file DGD-67-245-s013.tif]
