# Supplementary material for: Transcriptome Analysis Suggested Striking Transition Around the End of Epiboly in the Gene Regulatory Network Downstream of the Oct4‐Type POU Gene in Zebrafish Embryos
Source: Dev Growth Differ. 2025 Jun 9;67(5):245–69. doi: 10.1111/dgd.70012 (PMC12199784; doi:10.1111/dgd.70012)
Supplement: Supplementary file 2 — Figure S2. The sequence of the upstream DNA of pax2a from −3974 to +3 bp is shown with positions relative to the ATG codon on the right. The positions −3000, −2000, and − 1000 are shown with red letters. The noncoding conserved sequences (NCR‐1 and NCR‐2) are shown in light blue. The Distal and Proximal SOX‐POU elements are marked with underlines and the primer sequences used for PCR amplification are shown in yellow (Okuda et al. 2010). The SOX‐Pou5f3 binding site and TATA box identified previously are shown in green and gray, respectively (Onichtchouk et al. 2010). [file DGD-67-245-s015.docx]

Figure S2. Sequence of the upstream DNA of *her3.*

| CACGCAGTAATTAAAGATTCATGCCTCGTTTGAACTAAAACTGTATCCACGAGTCTTCAG -3915  TTCTTATACAAATTTGTTGCTTATTCTTTGCAGATACTTTGCAACTCAAAGAGCTTCAAT -3855  AGACACGATTTGGTGCTTAACAATGCGAGAGGTCTTTTGTCTTTCGTTTCGGACTGGATG -3795  GCTCTTCAAAGCTTACATCCTTCGCTGGGTTTCAAGTTACTATTCACTAATTTATTTCAG -3735  CCAAATACCCAAGCAGAATACTTAAATGTAATGATAATGTGCTATTATGTTTTTTCTGCA -3675  AGCCTAAGTCAAGCAAGACTTCTCAAATAGGAATTCGTGACTGTTGGGCCAGAAAAGTAT -3615  TTTCGCTTGGTGTTGATTTGTTATAACATAGACTTACAGTTCGTGGTTTGTCGTCTTCAT -3555  GACACCAAACACATTCGTAAGATCTTGTTTAAATTTATGGGGGGAAATGTGTTATTACAA -3495  TAATCGGCAAAGTTTTAGTTTTTTGGGAAAAAGGGTGCCTTGCGTCTTTTATTAGTTATC -3435  TGCCAGCACATAAGTAAATGGCAACTGTAAATAACAACACTAAAGCAATTTTACAGGATG -3375  AATAAGAAACAAAGCACTATTATAATCACTACATTATTTGTTTGCCTCATTGTCGTTCGA -3315  AAGATTGCAATGACCTTATGCCATTCAAGTCTTTCAGTTGTGCTGACAATTAAACGTGTG -3255  TTGATTATTTGTTTGACTTGCTTATACTCCTGGTAACTGTCATTCTGAGCTCAAGAACAT -3195  CGGCTGATTCTCTTTTACCGCAATTCTTTGCATTTACATGCTAAAAATGAATGGAAGCCC -3135  TTTGGAAAAGGAACGTTTATTGCAGTAATTGTATAATTGCTTCACTAATTGTTGTTAACA -3075  AGCCATAGAATGCTTCAGTCACTACGGGATCTCCTAACTAAGCGTGCATGATAAAAGGCA -3015  GTTAGGGTTAATCAGTTACAGATGGAGAATAATGACAGCACTTCCACGCTTTTGATAAAC -2955  ATGCGCTTTTATCGCAGTCTGTTGTTATGCCAAACTCGAAGACAATTTGTTCCCGTCAGA -2895  AAACCTTTCTAAATAAAGTGATATTCCATCTACATATTGTGTGTGTGCATTATTTATTTA -2835  TTTATTTATTTATCCATTTACTCTTTTATTCATTCATTCATTCATTCGTTCATTCATTCA -2775  GTTAGTTTGTTGGTTAGAAAAACTTTTGCGCAAATGACTAAAGATTTTCTGATGAAATCA -2715  AACTGTTAACCAGGGTGCCACTGAGCGTGAAGACGTGTAAATCAGTGTCACAAAAGAAAG -2655  CTGATTTGCTCCGATTTCATACTGATTTTCTAATGTTACGCTGTGAAAAGAGAAACACGG -2595  CAGCGTTTTTAACCAATGTTTTGCCCATTACAAACAATTCTCGAGATGCATGTACGACAT　-2535  GCATGGCTAACCTGTCTTTTAGTTTGTTAGCAGCCGAGAAAGTTTTCTTAAGTTCGGCCC -2475  AAAACCTTAAAAGGTTAATTGAAAATAGAGAACGACAGAAAGTCGTCAACAGGCTTCTTC -2415  ATTGTTTTCAAATAAATACTAAAGTTTTTAGTACAACTGTCCTGATTTTGCCATTAGCAA -2355  ATATTGTTTGCAAAGTTTGATGGAATATTGTCGTAATAGGACGGATTTATTTAGAAAAGA -2295  ACTCATTTCAGAAGATATACAATAGACCTGAAAGATCAATCGCTTCTCAATACATAGCCT -2235  ATATAAACTGCAGTTTTTCATTGCTTTCTAAAGGCTCTTGACCGGAGCTGCTAAAATGGT -2175  AATGTGATTCCATCCCGGTCTCACGTCATTTGTTCCCTCTCAATAAATACACTATTGTAT -2115  GGTAAGGCCGTCTCAACTGAAAAAAAAGTATTGAAAGACGAAAAAAAAGTCTTAAAGGAA -2055  TCAATGGACATTCATTCATCCTGTCGCGTGTCATGCTTTTGAAGTCATTTCTTACTACGC -1995  ATGTGCAGCAGGGCATTGTCATGTTGAAAAACGAGTTTGTTTTAGCAACAACAACAACAA -1935  CAACAAAAAATAAAAATAGATAAATAAAAAATAAATAAATAATATATAAAACATATTAAA -1875  ATAATAATATATATTGCTACAATATTATTTTCAAATTTTTTTTGCTTGACAATAGCGGAA -1815  CTGAAAATTCCAACTTGTGGAATTAAAAATTTAACTATTGAAAGGAAATCACGGTTAGCT -1755  TTTGAACTCGTCATCAATCATGAGCAATGAGAAATAGATATATTTTAAATACAACGGATT -1695  TCCACATCATCTCATATGGGTTACTCTTTTAAAATGGCTAACCTGCACCGTTTAGTTAAA -1635  CTGTTTTAAACTGATCCCGAATATCAGATCATTTATTTAGTGACTTAGTTCGTTTATTAT -1575  GTTAATCTTATAGCCACTGCACACCTGGCTATTTTTTGGAAGATAAAAAAGAAAGACATT -1515  GACTTGAGCTTTTTAAATGCATGAGGTCTGACATCTAAATTTTTTTGCGCAATATACAAA -1455  TGCATTTTCAGTTACCATTTTTGATAGGCTACTACAGCAGTAATGCAGTAAGCTAATGTA –1395  AAAAATCGGTGTCCCGAAACATTGTAACACTACTTTACCTAATTTAAAACTAAAGATTTA –1335  TATTTTTTTATAAAAAATAATCAATTGTATTGTGTTATCAATTGATTTACCTATTCGTAT –1275  CTTGTGTAAAAAATGACTAGTTTTAAGTAATATTTATTTTTAATTAAAATAAAATTGCCC –1215  CTGGGTTCCGCTATTTGTTTGCGTTTTTATTTGATTGGACGGTTGGCTGTTTAGTTAGTC -1155  AGCCAGTCAGTTAGTTAAGTAGTTAGGCTACCTATTAGTTATTTGTGGAAAAATATGTGA -1095  AACTTATGTGAAATATGTGCTTGTCAGCGAAGACTAGGCTGGAGGGAATTCACTTAAAAA -1035  GAAACTTCATTTTTGGCAAAAACCTGTATGACATTCCCAAGGCAGACTCTATTGATGGGA -975  AAGTGTGGAAAATCAGGCAGATAGGGTCCTGGGACAGGAGGCGTTCCTCTGATTCTCCTC -915  GCTCTGCTCTTTCAAGGGAGCATCTGTGAGAATTCTGCTTCTCTTTGAGACCATCTATAT -855  GTTATCAAGCTTAAACTTGTAGAGTTTTGCTTGTAGCACGACAGTCTGTATTCTACACAC -795  GGCCAGCGATTCCTAAAGTGTTTAGGCAGTTTTAGAAAATGTTGCTGTGGTTTAAAGAAT -735  CTTGACATTTTAATTCAGTAAAGGTGATTCCTTTTCATTATTGGCAGAAAATTGCCGCGT -675  TGTGAAGACACGGAAAAGATTTCCACGGTGCTGCCTGTCCCACATGGCGTGAAACCTGAA -615  GAGAATGGGATGAATAGTCACAGCTAATTCCTTCACAGCTTGACTAGTTATTTGTAACAT -555  TTTCCACTTTACGGTTTTTTACTGAACTCTTTGCATTTTCACCCCACACGACGAGGTTTC -495  AAGCCAGCGCCAGTTTAATTTTATCTAAGTATTAATACATGTAATAACAATTAAGTTTAC -435  AAGTCAGCAAAGAAAATTATATAATTAAAAATAGTTGTATAAAGTATTGATAGCATAATC -375  TATTATATTTTAAGTTCTGAAAATCTCTAATTAGTGACAAAACTAGTTTGTTTTGTGTTG -315  TCTACTTTTATGTATTAATTTTCATAGCGTATAAGAGTTTATAAAGTTTGTAAAAGATAG -255  CAGGACCAATATTATTTTTGAGCTCCTTTTTGAGAATAAAAACAGACAAGAGAGCAACCT -195  GAAGCTGATTGGATGTCCAGCAGAAAGTATGGATGCTCTTTTTTGAGCACCAATAATGTG -135  AGCAGTGCATTGACATGCTAATGGAGCTTTCCTTAAAAAGGAAAGAACTAGGGTGAATTC -75  CTTCACTCAACTCAAGGAAGTCTGATAGTCCTTATTCATCTGGATTTTTTAAACACACTG -15  AGCATTTAAGGACAATGNNNNNNNNNNNNNNNNNNNNNNNNNNNNNNNNNNNNNNNNNNN +3  NNNNNNNNNNNNNNNNNN NCR1/2 (this study)  NNNNNNNNNNNNNNNNNN Distal/Proximal SOX-POU elements (Okuda et al., 2010)  (Primers used for PCR are shown in yellow.)  CATTGACATGCTAAT SOX-Pou5f1 binding site (Onichtchouk et a., 2010)  TTAAAAA TATA site (Onichtchouk et a., 2010)  ATG start codon  N -1000 bp, -2000 bp, -3000 bp | NCR-1  Distal  NCR-2  Proximal |
| --- | --- |

Legend to Fig. S2

The sequence of the upstream DNA of *pax2a* from –3974 to +3 bp is shown with positions relative to the ATG codon on the right. The positions –3000, –2000, and –1000 are shown with red letters. The non-coding conserved sequences (NCR-1 and NCR-2) are shown in light blue. The Distal and Proximal SOX-POU elements are marked with underlines and the primer sequences used for PCR amplification are shown in yellow (Okuda et al., 2010). The SOX-Pou5f3 binding site and TATA box identified previously are shown in green and gray, respectively (Onichtchouk et al., 2010).
