# Supplementary material for: Transcriptome Analysis Suggested Striking Transition Around the End of Epiboly in the Gene Regulatory Network Downstream of the Oct4‐Type POU Gene in Zebrafish Embryos
Source: Dev Growth Differ. 2025 Jun 9;67(5):245–69. doi: 10.1111/dgd.70012 (PMC12199784; doi:10.1111/dgd.70012)
Supplement: Supplementary file 5 — Text S1. (Methods). [file DGD-67-245-s012.docx]

Supplementary Text (Methods)

*Plasmid constructs for luciferase assay*

For the luciferase assay, the 4.0-kb upstream region of zebrafish *her3* (cf. Fig. S2), which was previously shown to include the closely adjacent binding sites for Sox and POU (Okuda et al., 2010; Onichtchouk et al., 2010), was amplified by PCR from zebrafish genomic DNA and an appropriate primer pair (her3(–4.0)-f & her3-ATG-Bam-r; Table S2), and then ligated into the multicloning site (MCS) of pGL4.10[luc2], in which modified firefly luciferase, luc2, is employed as a reporter (referred to as pGL4 hereafter, Promega) (pHer3[–4.0]-Luc, cf. Fig. 5D). The same upstream 4.0-kb DNA was amplified by PCR using another primer pair (pHer3(–4.0)-f and her3-ATG-Apa-r; Table S2) and inserted into the MCS of pEGFP-1 (pHer3[–4.0]-EGFP) for *in vivo* reporter experiments.

Deletion of non-coding conserved regions (NCRs) 1 and/or 2 was conducted from pHer3[–4.0]-Luc by inverse PCR as below (cf. Fig. 5D). PCR was conducted using as a template pHer3[–4.0]-Luc DNA with appropriate inverse primer pairs flanking the target sequences to be deleted and tagged at the 5′-ends with restriction sequences (her3-NCR1-SphI-f and her3-NCR1-SphI-r; her3-NCR2-NheI-f and her3-NCR2-NheI-r, Table S2). After digestion with appropriate enzymes, the PCR products were self-ligated to produce deleted constructs (pHer3[–4.0]dNCR1-Luc, pHer3[–4.0]dNCR2-Luc). To delete both NCR sequences, pHer3–4.0]dNCR2-Luc was used as a template to delete NCR-1 as described above (pHer3[–4.0]dNCRs-Luc).

The coding regions of regulatory genes were amplified by reverse-transcription PCR of total RNA from embryos and ligated into pCS2+ (Table S2), which were used as effector genes in luciferase assays. The expression of the genes was driven in transfected cells by the upstream cytomegalovirus promoter. For control, pCS2+ harboring the *enhanced green fluorescent protein* (*egfp*) gene (pCS2+egfp) was used instead.

For synthesis of *en-pou5f3*-ERT2 mRNA, the template plasmid was built; the detail will be available on request (cf. Fig. 4A). Briefly, the coding sequence of *en-pou5f3* lacking the stop codon was amplified by PCR and replaced with the KalTA4 sequence in pKalTA4-ERT2 (kindly donated by Dr. M. Tada), resulting in a pCS2+-based plasmid in which the complete *en-pou5f3* coding sequence was fused to the ERT2 sequence (pCS2+en-pou5f3-ERT2).

PCR for plasmid construction was conducted with high-fidelity DNA polymerase (LA Taq polymerase, TaKaRa), and the structures of the constructs were verified by sequencing.

*Microarray analysis.*

Embryos from crosses between *en-pou5f3^+/–^* and wild-type fish were subjected at 90% epiboly or 3-ss to heat shock as described above. After a 30-min interval at normal culture temperature, genomic DNA and total RNA were separately extracted from 10–12 individual embryos, and respective heat-treated embryos were genotyped using the genomic DNA. RNA was purified from the pooled extracts combined based on the genotype (>5 embryos/pool). To reduce variance in quality among samples, RNAs were prepared by a single experimenter from differently treated single-clutch embryos on the same day, using reagents of the same lots and reagent master mixes. The quality of purified RNA was evaluated in an Agilent Bioanalyzer 2100, using the RNA 6000 nano Assay Kit. Purified RNA was subjected to the microarray analysis (KURABO, Osaka, Japan) using the Affymetrix GeneChip® Zebrafish Genome Array (15,617 probes, Affymetrix, Santa Clara, CA, USA) to comprehensively compare the transcriptome between *en-pou5f3^+/–^* and wild-type embryos (hereafter referred to as ‘*en-pou5f3+*’ and ‘sibling’ embryos, respectively) employing the single comparison analysis following the protocol provided by Agilent (<https://assets.thermofisher.com/TFS-Assets/LSG/manuals/data_analysis_fundamentals_manual.pdf>), as was conducted before (Nakayama et al., 2017) (Table S3). It should be mentioned that the term ‘probe’ is correct, but for convenience’s sake, the word ‘gene’ will be frequently used instead hereafter. The signal values for probe sets from 12 arrays are given (6 control and 6 experimental)*.* The Affymetrix GeneChip® Operating Software (GCOS) was used to determine signal intensities and detection calls for each gene (cut off *p*-values; α1=0.05, α2=0.065, Tau=0.015). Alteration of the expression levels was tested by the Wilcoxon Signed Rank Test and judged as statistically significant using *p*=0.0025 as a cut-off value (increase, *p* < 0.0025; decrease, *p* > 0.9975). When more severe criteria were necessary, the data were extracted when the fold change is more than twice or less than half. Gene ontology (GO) analysis (Aleksander et al., 2023), Pathway analysis (García-Campos et al., 2015), and InterPro analysis (Paysan-Lafosse et al., 2023) were performed based on these data using DAVID Bioinformatics Resources 6.7 (<https://david.ncifcrf.gov>) for GO analysis, Pathway analysis, and InterPro analysis and DAVID 6.8 (<https://davidbioinformatics.nih.gov/summary.jsp>) for Functional Annotation Clustering analysis (Sherman et al., 2022). The significance of the association was examined by the Fisher’s exact test. The primary microarray data generated in this study have been submitted to GEO (http:// [www.ncbi.nlm.nih.gov/geo/)(GSE271502](http://www.ncbi.nlm.nih.gov/geo/)(GSE271502), GSM8378058, GSM8378059, GSM8378060, GSM8378061).
