# Supplementary material for: Transcriptome Analysis Suggested Striking Transition Around the End of Epiboly in the Gene Regulatory Network Downstream of the Oct4‐Type POU Gene in Zebrafish Embryos
Source: Dev Growth Differ. 2025 Jun 9;67(5):245–69. doi: 10.1111/dgd.70012 (PMC12199784; doi:10.1111/dgd.70012)
Supplement: Supplementary file 6 — Table S1. [file DGD-67-245-s016.docx]

Table S1. Oligonucleotides used in the current study for qRT-PCR^1^.

| Primer name^1^ | Sequence (5′ → 3′) | Size (nucleotides) |
| --- | --- | --- |
| cops4-q-f | CCACCACAGCCGATGGTTC | 19 |
| cops4-q-r | TGGTCAATGAATCCGTTCATGC | 22 |
| her3-q-f | TCCGCAAGCGAAAACTGGAA | 20 |
| her3-q-r | GCATGATGGAGCGGGAATCT | 20 |
| hesx1-q-f | ACAGCCTTCTCCAGTGTTCA | 20 |
| hesx1-q-r | AACTGGGACTCTCTGTGCGA | 20 |
| kif5aa-q-f | CCGCGACAGTAAGATGACCA | 20 |
| kif5aa-q-r | CAGGTTAATGGAGGCCGTGT | 20 |
| lrrtm1-q-f | TTGCGGGAAGGGAACTTTGT | 20 |
| lrrtm1-q-r | GGTCTAAAGGTCCCGTTGGG | 20 |
| otx1a-q-f | AAAGGTGTCTCTCTGGGGCT | 20 |
| otx1a-q-r | AAGATGTCCAGCTGAGTGCG | 20 |
| pax6b-q-f | AACCAGAGACGACAAGCCAG | 20 |
| pax6b-q-r | AGCTGGGCATTGGTGGTAAA | 20 |
| pth2r-q-f | CAGGAAGCTTGCCAAATCCAC | 21 |
| pth2r-q-r | ATCTCAGTCTGGACCTCACC | 20 |
| slc16a9a-q-f | CTCATCACTAGCCCGGTGTG | 20 |
| slc16a9a-q-r | TGGTCAGTGTGGCAGCATAG | 20 |
| sox21b-q-f | CGTTCCCGGTGGCGTATAAT | 20 |
| sox21b-q-r | TACGGGTTTGCGGACATTGA | 20 |
| sox9b-q-f | CAGAAACACCCGACTCCAGC | 20 |
| sox9b-q-r | CCGAAGTCAATGCCGTCCT | 19 |
| stxbp6l-q-f | ATCTGGGCTCCAGGAAGGAA | 20 |
| stxbp6l-q-r | AGGGAAAGTCCCGTCCTCTT | 20 |
| tbx2b-q-f | GTCACACTGGAAGCGAAGGA | 20 |
| tbx2b-q-r | AAACTTGTAGCGGCAGTCGT | 20 |
| zic4-q-f | ATGTCCGCGAGAAGGAAAGC | 20 |
| zic4-q-r | TCACAGCCGTCAAACTCACA | 20 |
| luc2-q-s | CACCTTCGTGACTTCCCATTTG | 23 |
| luc2-q-as | TGACTGAATCGGACACAAGCGG | 22 |
| renilla-luc-q-s | CTGATCTGATCGGAATGGGT | 20 |
| renilla-luc-q-as | TGATCTTGTCTTGGTGCTCG | 20 |

1. For each gene, a sense and an antisense primer were designed for quantitation of mRNA by qRT-PCR. ‘f’ and ‘r’ show sense (forward) and reverse (antisense) primers, respectively. ‘q’ shows primers for qRT-PCR.
