# Supplementary material for: Transcriptome Analysis Suggested Striking Transition Around the End of Epiboly in the Gene Regulatory Network Downstream of the Oct4‐Type POU Gene in Zebrafish Embryos
Source: Dev Growth Differ. 2025 Jun 9;67(5):245–69. doi: 10.1111/dgd.70012 (PMC12199784; doi:10.1111/dgd.70012)
Supplement: Supplementary file 7 — Table S2. [file DGD-67-245-s020.docx]

Table S2. Oligonucleotides used in the current study for cloning of cDNA and genomic DNA by PCR amplification.

| Purpose | Primer name^1^ | Sequence (5′ → 3′)^2^ | Size (nucleotides) |
| --- | --- | --- | --- |
| cDNA cloning^3^ | barhl2-RI-f | GCGAATTCTTCTTATACTATGGAAGGATCC | 30 |
|  | barhl2-Xb-r | GCTCTAGATCTTCTTGTTTTACCGAGCAT | 29 |
|  | cops4-RI-f | GCGAATTCTGCGGAGGAAAATGGCGTCC | 28 |
|  | cops4-XhoI-r | GGCCTCGAGATAAGGTTTATTGGGACATCTGG | 32 |
|  | hhatlb-RI-f | GCGAATTCCAAGGTGGATAATGGGGGTCAA | 30 |
|  | hhatlb-Xb-r | GCTCTAGATGAGTGTCTCTACTCTGCCTTT | 30 |
|  | kif5aa-RI-f | GCGAATTCTTCTGCATCATCATGACGGAC | 29 |
|  | kif5aa-Xb-r | GCTCTAGATTAAATGATTAACTGGCTGCAG | 30 |
|  | lect1-f | TTCACGCAGGAACGCCATGGA | 21 |
|  | lect1-r | AAGCGTCTGGATCTCCTACACCA | 23 |
|  | lrrtm1-f | GCAGGATATTCATGCTAATGG | 21 |
|  | lrrtm1-r | GGGTGGAATATCACACTTCG | 20 |
|  | pou3f3a-RI-f | GCGAATTCAGATGATGAGGAGGGATGGCCA | 30 |
|  | pou3f3a-Xb-r | GCTCTAGAGTTTTCGGTGTCAGTGCACCAG | 30 |
|  | pth2r-RI-f | GCGAATTCTTGATCTTCAGGATGCTGACG | 29 |
|  | pth2r-Xb-r | GCTCTAGACCACATCGTTATCTACAGAGTT | 30 |
|  | sebox-RI-f | GCGAATTCGAATCTGGACTATGGCGCTAT | 29 |
|  | sebox-RI-r | GCTCTAGAGTCTCCAGTGTTTAGCACTCT | 29 |
|  | slc20a1a-f | TTCTCCAGAAATGGAATCCACT | 22 |
|  | slc20a1a-r | AATGTAACGCATTAGGTCAGCG | 22 |
|  | sox21b-RI-f | GCGAATTCAGGTGAACGCGTCATGTCCAA | 29 |
|  | sox21b-Xb-r | GCTCTAGATTAGAGTCTCATAACGCCGCC | 29 |
|  | sox9b-RI-f | GCGAATTCCTCTCTCTGCATGAATCTCCTCCA | 32 |
|  | sox9b-Xb-r | GCTCTAGATGTGTGCTCAGGGTCTGGACA | 29 |
|  | stxbp6l-f | GGCATTTTGAAGATGAACATTC | 22 |
|  | stxbp6l-r | CAGAATACCTTTAGATCTGC | 20 |
|  | zic4-RI-f | GCGAATTCCTGGGGAGATGACTGCGGAA | 28 |
|  | zic4-Xb-r | GCTCTAGACAATGTGTTAGAAGGCGTCCCT | 30 |
| Construct^4^ | her3(-4.0)-f | GCGTCGACCACCGCAGTAATTAAAGATTCATGCC | 34 |
|  | her3-ATG-Bam-r | GCGGATCCTGTCCTTAAATGCTCAGTGTGT | 30 |
|  | her3-ATG-Apa-r | GGCGGGCCCTGTCCTTAAATGCTCAGTGTG | 30 |
|  | her3-NCR1-SphI-f | GCGCATGCCGTGCATGATAAAAGGCAGT | 28 |
|  | her3-NCR1-SphI-r | GCGCATGCTGTAAATGCAAAGAATTGCGG | 29 |
|  | her3-NCR2-NheI-f | GCGCTAGCTCACAGCTAATTCCTTCACA | 28 |
|  | her3-NCR2-NheI-r | GCGCTAGCATGAAAAGGAATCACCTTTAC | 29 |

1. ‘f’ and ‘r’ represent forward (sense) and reverse (antisense) primers.
2. Target sequences of restriction enzymes shown in the oligo names are underlined. Apa, *Apa*I; RI, *Eco*RI; Xb, *Xba*I; Bam, *Bam*HI; Apa, *Apa*I.
3. Oligos used for cDNA cloning.
4. Oligos used for building plasmid constructs.
