# Supplementary material for: Transcriptome Analysis Suggested Striking Transition Around the End of Epiboly in the Gene Regulatory Network Downstream of the Oct4‐Type POU Gene in Zebrafish Embryos
Source: Dev Growth Differ. 2025 Jun 9;67(5):245–69. doi: 10.1111/dgd.70012 (PMC12199784; doi:10.1111/dgd.70012)
Supplement: Supplementary file 13 — Table S8. [file DGD-67-245-s017.docx]

Table S8. Genes significantly downregulated by *en-pou5f3* at both stages of induction^1^

| Gene Title | Gene Symbol | 90% epiboly, Signal Log Ratio | 3-somite stage, Signal Log Ratio |
| --- | --- | --- | --- |
| *hairy-related 3* | *her3* | -11 | -8.4 |
| *paired box gene 6b* | *pax6b* | -6.2 | -2.1 |
| *homeo box (expressed in ES cells) 1* | *hesx1* | -5.1 | -1.8 |
| *orthodenticle homolog 1a* | *otx1a* | -5.1 | -1.2 |
| *forkhead box B1.2* | *foxb1.2* | -5.0 | -3.2 |
| *iroquois homeobox protein 1, a* | *irx1a* | -4.8 | -1.9 |
| *mix-type homeobox gene 1* | *mxtx1* | -4.5 | -2.2 |
| *zic family member 1 (odd-paired homolog, Drosophila)* | *zic1* | -4.4 | -2.2 |
| *orthodenticle homolog 1b /// wu:fc92e03* | *otx1b /// wu:fc92e03* | -4.3 | -2.7 |
| *Wnt8-like protein 2-like /// wnt8-like protein 2 /// wingless-type MMTV integration site family, member 8a* | *LOC100329734 /// wnt8-2 /// wnt8a* | -4.1 | -2.1 |
| *goosecoid* | *gsc* | -4.1 | -1.9 |
| *Sp5 transcription factor-like* | *sp5l* | -4.1 | -3.1 |
| *---* | *---* | -4.1 | -3.2 |
| *putative transmembrane protein TA-2-like* | *LOC565377* | -3.8 | -2.1 |
| *zic family member 4* | *zic4* | -3.6 | -4.8 |
| *neurogenin 1* | *neurog1* | -3.5 | -3.0 |
| *forkhead box D5* | *foxd5* | -3.3 | -2.2 |
| *protocadherin 18a* | *pcdh18a* | -3.2 | -1.8 |
| *one-eyed pinhead* | *oep* | -3.1 | -1.5 |
| *sine oculis homeobox homolog 7* | *six7* | -3.1 | -3.6 |
| *SRY-box containing gene 2* | *sox2* | -3.1 | -1.1 |
| *v-maf musculoaponeurotic fibrosarcoma (avian) oncogene homolog* | *maf* | -3.0 | -1.3 |
| *posterior neuron-specific homeobox* | *pnx* | -3.0 | -1.2 |
| *sal-like 4 (Drosophila)* | *sall4* | -2.8 | -2.0 |
| *rad and gem related GTP binding protein 1* | *rem1* | -2.7 | -2.1 |
| *forkhead box B1.1* | *foxb1.1* | -2.6 | -1.8 |
| *paired box gene 2a* | *pax2a* | -2.6 | -1.1 |
| *wu:fe11c03* | *wu:fe11c03* | -2.6 | -1.1 |
| *frizzled homolog 8b* | *fzd8b* | -2.5 | -2.8 |
| *transcription factor AP-2 alpha* | *tfap2a* | -2.5 | -2.0 |
| *forkhead box D3* | *foxd3* | -2.5 | -1.7 |
| *fibroblast growth factor 8 a* | *fgf8a* | -2.4 | -2.5 |
| *---* | *---* | -2.4 | -4.8 |
| *PDZ domain-containing protein 2-like* | *LOC100149069* | -2.4 | -1.5 |
| *SRY-box containing gene 11b* | *sox11b* | -2.3 | -1.6 |
| *BarH-like 2* | *barhl2* | -2.3 | -3.9 |
| *LIM homeobox 5* | *lhx5* | -2.2 | -1.2 |
| *protocadherin 8* | *pcdh8* | -2.2 | -1.5 |
| *SET and MYND domain containing 4* | *smyd4* | -2.2 | -1.5 |
| *Butyrate response factor 1-like* | *LOC100334443* | -2.1 | -1.5 |
| *SRY-box containing gene 11b* | *sox11b* | -2.0 | -1.5 |
| *paired-like homeodomain transcription factor 2* | *pitx2* | -2.0 | -1.8 |
| *induced in neural crest by AP2, 1b* | *inka1b* | -2.0 | -2.0 |
| *induced in neural crest by AP2, 1a* | *inka1a* | -2.0 | -1.0 |
| *---* | *---* | -2.0 | -2.4 |
| *iroquois homeobox protein 7* | *irx7* | -1.9 | -1.6 |
| *hairy-related 5* | *her5* | -1.9 | -2.6 |
| *SRY-box containing gene 3* | *sox3* | -1.9 | -1.5 |
| *wu:fb39e08* | *wu:fb39e08* | -1.9 | -2.1 |
| *RGM domain family, member A* | *rgma* | -1.8 | -1.2 |
| *wu:fd23c12* | *wu:fd23c12* | -1.8 | -2.0 |
| *induced in neural crest by AP2, 1a* | *inka1a* | -1.8 | -1.5 |
| *retinal homeobox gene 3* | *rx3* | -1.7 | -1.8 |
| *SRY-box containing gene 21 b* | *sox21b* | -1.7 | -4.9 |
| *chordin* | *chd* | -1.6 | -1.6 |
| *sonic hedgehog b* | *shhb* | -1.6 | -2.3 |
| *protocadherin 10b* | *pcdh10b* | -1.6 | -1.2 |
| *myelocytomatosis oncogene homolog* | *mych* | -1.6 | -1.2 |
| *sp8 transcription factor b* | *sp8b* | -1.6 | -1.1 |
| *Leucine rich repeat transmembrane neuronal 1* | *lrrtm1* | -1.6 | -4.0 |
| *SRY-box containing gene 11a* | *sox11a* | -1.5 | -1.0 |
| *achaete-scute complex-like 1a (Drosophila)* | *ascl1a* | -1.5 | -1.2 |
| *iroquois homeobox protein 1, b* | *irx1b* | -1.5 | -1.7 |
| *SRY-box containing gene 3* | *sox3* | -1.5 | -1.3 |
| *zgc:153310* | *zgc:153310* | -1.5 | -1.7 |
| *v-myc myelocytomatosis viral related oncogene, neuroblastoma derived (avian)* | *mycn* | -1.5 | -1.5 |
| *Kruppel-like factor 4* | *klf4* | -1.4 | -1.7 |
| *myogenic factor 5* | *myf5* | -1.4 | -1.0 |
| *solute carrier family 25 (mitochondrial carrier: glutamate), member 22* | *slc25a22* | -1.4 | -1.4 |
| *regulator of G-protein signaling 2* | *rgs2* | -1.4 | -2.4 |
| *SRY-box containing gene 11a* | *sox11a* | -1.3 | -1.2 |
| *homeo box C6b* | *hoxc6b* | -1.3 | -1.2 |
| *aquaporin 3a* | *aqp3a* | -1.3 | -1.3 |
| *GATA-binding protein 2a* | *gata2a* | -1.3 | -1.1 |
| *eph receptor A2* | *epha2* | -1.3 | -1 |
| *LIM homeobox 1a* | *lhx1a* | -1.2 | -1.1 |
| *---* | *---* | -1.2 | -1.2 |
| *v-myc myelocytomatosis viral related oncogene, neuroblastoma derived (avian)* | *mycn* | -1.2 | -1.3 |
| *ATP-binding cassette, sub-family B (MDR/TAP), member 5* | *abcb5* | -1.2 | -1.1 |
| *POU class 2 homeobox 1b* | *pou2f1b* | -1.2 | -1.3 |
| *chemokine (C-X-C motif) receptor 7b* | *cxcr7b* | -1.2 | -1.4 |
| *diencephalon/mesencephalon homeobox 1a* | *dmbx1a* | -1.1 | -2 |
| *FEZ family zinc finger 2* | *fezf2* | -1.1 | -1 |
| *mesoderm posterior b* | *mespb* | -1.1 | -1.3 |
| *sine oculis homeobox homolog 3b* | *six3b* | -1.1 | -1.3 |
| *bone morphogenetic protein receptor, type 1ba* | *bmpr1ba* | -1.1 | -1 |
| *protein tyrosine phosphatase, receptor type, N polypeptide 2* | *ptprn2* | -1.1 | -1 |
| *wu:fb24e03* | *wu:fb24e03* | -1.1 | -1.2 |
| *zgc:154020* | *zgc:154020* | -1.1 | -2.6 |
| *sine oculis homeobox homolog 3a* | *six3a* | -1 | -1.4 |
| *Kruppel-like factor 3 (basic)* | *klf3* | -1 | -1.2 |
| *cugbp, Elav-like family member 3* | *celf3* | -1 | -1.3 |
| *zgc:162730* | *zgc:162730* | -1 | -1.2 |
| *transcription factor AP-2 gamma (activating enhancer binding protein 2 gamma)* | *tfap2c* | -1 | -1.3 |
| *es1 protein-like* | *LOC565309* | -1 | -1.1 |
| *POU class 2 homeobox 1b* | *pou2f1b* | -1 | -1 |

1. Genes are listed when signal log ratios for alterations of mRNA levels due to *en-pou5f3* induction were -1 or less (two-fold or more decrease) at both 90% epiboly and the 3-somite stage and ordered ascengingly. Signal log ratios represent the logaristhms of the ratios of *en-pou5f3*-induced expressions to controls.
