# Supplementary material for: Transcriptome Analysis Suggested Striking Transition Around the End of Epiboly in the Gene Regulatory Network Downstream of the Oct4‐Type POU Gene in Zebrafish Embryos
Source: Dev Growth Differ. 2025 Jun 9;67(5):245–69. doi: 10.1111/dgd.70012 (PMC12199784; doi:10.1111/dgd.70012)
Supplement: Supplementary file 14 — Table S9. [file DGD-67-245-s002.docx]

Table S9. Genes significantly upregulated at both stages examined by *en-pou5f3* induction^1^

| Gene Title | Gene Symbol | 90% epiboly, Signal Log Ratio | 3-somite stage, Signal Log Ratio |
| --- | --- | --- | --- |
| *---* | *---* | 5.3 | 3.2 |
| *hypothetical LOC798783* | *LOC798783* | 2.2 | 1.4 |
| *thioredoxin interacting protein b* | *txnipb* | 2.0 | 1.1 |
| *interferon regulatory factor 11* | *irf11* | 1.9 | 1.2 |
| *E74-like factor 3 (ets domain transcription factor, epithelial-specific )* | *elf3* | 1.6 | 1.7 |
| *---* | *---* | 1.5 | 1.0 |
| *chemokine (C-X-C motif) ligand 14* | *cxcl14* | 1.4 | 1.0 |
| *wu:fc22g01* | *wu:fc22g01* | 1.4 | 5.0 |
| *---* | *---* | 1.4 | 1.3 |
| *---* | *---* | 1.4 | 1.0 |
| *vessel-specific 1* | *vsg1* | 1.3 | 1.5 |
| *---* | *---* | 1.3 | 1.1 |
| *granulin 1* | *grn1* | 1.2 | 1.6 |
| *tocopherol (alpha) transfer protein (ataxia (Friedreich-like) with vitamin E deficiency)* | *ttpa* | 1.2 | 1.3 |
| *granulin 1 /// granulin 2* | *grn1 /// grn2* | 1.1 | 1.9 |
| *zgc:198406* | *zgc:198406* | 1.1 | 2.2 |
| *kinesin family member 5A* | *kif5a* | 1.1 | 4.5 |
| *wu:fe16d09* | *wu:fe16d09* | 1.1 | 1.4 |
| *Si:dkey-127j5.5* | *si:dkey-127j5.5* | 1.1 | 1.5 |
| *ST3 beta-galactoside alpha-2,3-sialyltransferase 3* | *st3gal3* | 1.0 | 1.0 |
| *protein tyrosine phosphatase, receptor type, N polypeptide 2, like* | *ptprn2l* | 1.0 | 2.1 |
| *---* | *---* | 1.0 | 1.6 |
| *zgc:85866* | *zgc:85866* | 1.0 | 1.4 |
| *---* | *---* | 1.0 | 1.9 |

1. Genes are listed when signal log ratios for alterations of mRNA levels due to *en-pou5f3* induction were one or more (two-fold or more increase) at both 90% epiboly and the 3-somite stage and ordered descengingly. Signal log ratios represent the logaristhms of the ratios of *en-pou5f3*-induced expressions to controls.
