# Supplementary material for: Transcriptome Analysis Suggested Striking Transition Around the End of Epiboly in the Gene Regulatory Network Downstream of the Oct4‐Type POU Gene in Zebrafish Embryos
Source: Dev Growth Differ. 2025 Jun 9;67(5):245–69. doi: 10.1111/dgd.70012 (PMC12199784; doi:10.1111/dgd.70012)
Supplement: Supplementary file 19 — Table S14. [file DGD-67-245-s006.docx]

Table S14. Clustering of genes based on the GO analysis.^1^

A. Genes significantly downregulated at 90% epiboly by *en-pou5f3* induction (Group I).

| Transcriptional Regulation | | | |
| --- | --- | --- | --- |
| Annotation | Term | Count | *p*-value |
| GO-MF | Transcription regulator activity | 113 | 4.3x10^–23^ |
| GO-MF | DNA binding | 129 | 9.6x10^–21^ |
| GO-BP | Regulation of transcription | 128 | 2.1x10^–20^ |
| GO-MF | Transcription factor activity | 91 | 2.2x10^–20^ |
| GO-MF | Sequence-specific DNA binding | 76 | 4.5x10^–18^ |
| GO-BP | Regulation of RNA metabolic process | 103 | 7.1x10^–18^ |
| GO-BP | Regulation of transcription, DNA-dependent | 102 | 1.0x10^–17^ |

| Developmental Regulation | | | |
| --- | --- | --- | --- |
| Annotation | Term | Count | *p*-value |
| GO-BP | Regionalization | 44 | 7.7x10^–18^ |
| GO-BP | Pattern specification process | 48 | 1.7x10^–15^ |
| GO-BP | Anterior/posterior pattern formation | 30 | 7.9x10^–13^ |
| GO-BP | Segmentation | 20 | 8.4x10^–9^ |
| GO-BP | Somitogenesis | 19 | 1.0x10^–8^ |
| GO-BP | Chordate embryonic development | 25 | 6.0x10^–8^ |
| GO-BP | Embryonic development ending in birth or egg hatching | 25 | 6.0x10^–8^ |
| GO-BP | Dorsal/ventral pattern formation | 18 | 8.4x10^–8^ |

| Cell Motility | | | |
| --- | --- | --- | --- |
| Annotation | Term | Count | *p*-value |
| GO-BP | Embryonic morphogenesis | 39 | 7.8x10^–11^ |
| GO-BP | Cell migration | 23 | 3.9x10^–7^ |
| GO-BP | Cell motility | 23 | 2.5x10^–6^ |
| GO-BP | Localization of cell | 23 | 2.5x10^–6^ |
| GO-BP | Ameboidal cell migration | 18 | 4.9x10^–6^ |
| GO-BP | Cell motion | 25 | 5.6x10^–6^ |
| GO-BP | Gastrulation | 19 | 1.7x10^–5^ |
| GO-BP | Cell migration involved in gastrulation | 1814 | 3.3x10^–5^ |

B. Genes significantly upregulated at 90% epiboly by *en-pou5f3* induction (Group II).

| Transcriptional Regulation | | | |
| --- | --- | --- | --- |
| Annotation | Term | Count | *p*-value |
| GO-BP | Regulation of transcription | 76 | 1.9x10^–6^ |
| GO-MF | Transcription regulator activity | 64 | 3.8x10^–6^ |
| GO-MF | DNA binding | 78 | 5.6x10^–6^ |
| GO-MF | Transcription factor activity | 44 | 1.2x10^–3^ |
| GO-BP | Regulation of transcription, DNA-dependent | 52 | 1.2x10^–3^ |
| GO-MF | Sequence-specific DNA binding | 37 | 1.3x10^–3^ |
| GO-BP | Regulation of RNA metabolic process | 52 | 1.6x10^–3^ |

| Developmental Regulation | | | |
| --- | --- | --- | --- |
| Annotation | Term | Count | *p*-value |
| GO-BP | Embryonic morphogenesis | 21 | 8.4x10^–4^ |
| GO-BP | Embryonic organ morphogenesis | 11 | 1.9x10^–3^ |
| GO-BP | Sensory organ development | 18 | 5.1x10^–3^ |
| GO-BP | Otic vesicle formation | 5 | 7.7x10^–3^ |
| GO-BP | Embryonic organ development | 13 | 1.6x10^–2^ |
| GO-BP | Inner ear morphogenesis | 6 | 2.0x10^–2^ |
| GO-BP | Ear morphogenesis | 6 | 2.2x10^–2^ |
| GO-BP | Otic placode formation | 4 | 2.5x10^–2^ |
| GO-BP | Inner ear development | 7 | 5.1x10^–2^ |
| GO-BP | Ear development | 7 | 5.4x10^–2^ |

| Chromatin-Chromosome | | | |
| --- | --- | --- | --- |
| Annotation | Term | Count | *p*-value |
| GO-BP | Cellular macromolecular complex assembly | 13 | 6.3x10^–4^ |
| GO-CC | Nucleosome | 7 | 8.8x10^–4^ |
| GO-CC | Protein-DNA complex | 7 | 1.2x10^–3^ |
| GO-BP | Cellular macromolecular complex subunit organization | 13 | 1.5x10^–3^ |
| GO-BP | Nucleosome assembly | 7 | 3.4x10^–3^ |
| GO-BP | Nucleosome organization | 7 | 3.4x10^–3^ |
| GO-BP | Protein-DNA complex assembly | 7 | 3.4x10^–3^ |
| GO-BP | Chromatin assembly | 7 | 3.4x10^–3^ |
| GO-BP | DNA packaging | 7 | 6.5x10^–3^ |
| GO-CC | Chromatin | 8 | 8.9x10^–3^ |
| GO-BP | Macromolecular complex assembly | 13 | 9.7x10^–3^ |
| GO-CC | Chromosomal part | 10 | 1.0x10^–2^ |
| GO-BP | Macromolecular complex subunit organization | 13 | 1.4x10^–2^ |
| GO-BP | Chromatin assembly or disassembly | 8 | 1.5x10^–2^ |
| GO-CC | Chromosome | 10 | 4.9x10^–2^ |
| GO-BP | Cellular protein complex assembly | 6 | 6.3x10^–2^ |
| GO-BP | Chromatin organization | 10 | 6.8x10^–2^ |
| GO-BP | Chromosome organization | 11 | 7.1x10^–2^ |
| GO-BP | Protein polymerization | 3 | 2.2x10^–1^ |
| GO-BP | Protein complex assembly | 6 | 3.1x10^–1^ |
| GO-BP | Protein complex biogenesis | 6 | 3.1x10^–1^ |
| GO-MF | GTPase activity | 3 | 7.7x10^–1^ |

C. Genes significantly downregulated at the 3-somite stage by *en-pou5f3* induction (Group III).

| Transcriptional Regulation | | | | |  |
| --- | --- | --- | --- | --- | --- |
| Annotation | Term | Count | | *p*-value |  |
| GO-BP | Regulation of transcription | 142 | 1.3x10^–29^ | | |
| GO-MF | Transcription regulator activity | 122 | 3.7x10^–28^ | | |
| GO-MF | DNA binding | 142 | 2.4x10^–27^ | | |
| GO-MF | Transcription factor activity | 96 | 3.3x10^–23^ | | |
| GO-BP | Regulation of RNA metabolic process | 109 | 2.7x10^–22^ | | |
| GO-BP | Regulation of transcription, DNA-dependent | 108 | 4.0x10^–22^ | | |
| GO-BP | Sequence-specific DNA binding | 82 | 1.4x10^–21^ | | |

| Developmental regulation | | | |
| --- | --- | --- | --- |
| Annotation | Term | Count | *p*-value |
| GO-BP | Regionalization | 32 | 1.2x10^–9^ |
| GO-BP | Anterior/posterior pattern formation | 25 | 2.4x10^–9^ |
| GO-BP | Pattern specification process | 36 | 1.3x10^–8^ |
| GO-BP | Chordate embryonic development | 22 | 2.2x10^–6^ |
| GO-BP | Embryonic development ending in birth or egg hatching | 22 | 2.2x10^–6^ |
| GO-BP | Somitogenesis | 14 | 3.7x10^–5^ |
| GO-BP | Segmentation | 14 | 1.0x10^–4^ |

| Neuronal Development | | | |
| --- | --- | --- | --- |
| Annotation | Term | Count | *p*-value |
| GO-BP | Embryonic morphogenesis | 42 | 3.0x10^–13^ |
| GO-BP | Embryonic organ development | 29 | 6.6x10^–10^ |
| GO-BP | Sensory organ development | 33 | 1.3x10^–8^ |
| GO-BP | Embryonic organ morphogenesis | 17 | 2.8x10^–6^ |
| GO-BP | Inner ear morphogenesis | 10 | 1.0x10^–4^ |
| GO-BP | Hindbrain morphogenesis | 6 | 1.1x10^–4^ |
| GO-BP | Ear morphogenesis | 10 | 1.3x10^–4^ |
| GO-BP | Inner ear development | 10 | 5.4x10^–3^ |
| GO-BP | Ear development | 10 | 6.0x10^–3^ |
| GO-BP | Otic placode formation | 5 | 7.0x10^–3^ |
| GO-BP | Semicircular canal morphogenesis | 4 | 1.5x10^–2^ |
| GO-BP | Otic vesicle formation | 5 | 1.6x10^–2^ |

D. Genes significantly upregulated at the e-somite stage by *en-pou5f3* induction (Group IV).

| Chromatin-Chromosome | | | |
| --- | --- | --- | --- |
| Annotation | Term | Count | *p*-value |
| GO-CC | Nucleosome | 7 | 5.8x10^–5^ |
| GO-CC | Protein-DNA complex | 7 | 8.3x10^–5^ |
| GO-BP | Cellular macromolecular complex assembly | 11 | 3.6x10^–4^ |
| GO-BP | macromolecular complex assembly | 13 | 3.6x10^–4^ |
| GO-CC | Chromatin | 8 | 5.1x10^–4^ |
| GO-BP | Macromolecular complex subunit organization | 13 | 5.7x10^–4^ |
| GO-BP | Cellular macromolecular complex subunit organization | 11 | 7.8x10^–4^ |
| GO-BP | DNA packaging | 7 | 9.1x10^–4^ |
| GO-BP | Nucleosome organization | 6 | 3.2x10^–3^ |
| GO-BP | Chromatin assembly | 6 | 3.2x10^–3^ |
| GO-BP | nucleosome assembly | 6 | 3.2x10^–3^ |
| GO-BP | Protein-DNA complex assembly | 6 | 3.2x10^–3^ |
| GO-CC | Chromosomal part | 8 | 6.3x10^–3^ |
| GO-CC | Chromosome | 8 | 2.5x10^–2^ |
| GO-BP | Chromatin assembly or disassembly | 6 | 3.1x10^–2^ |
| GO-BP | Chromosome organization | 8 | 1.0x10^–1^ |
| GO-BP | Protein complex biogenesis | 6 | 1.0x10^–1^ |
| GO-BP | Protein complex assembly | 6 | 1.0x10^–1^ |
| GO-BP | Chromatin organization | 7 | 1.3x10^–1^ |
| GO-CC | Intracellular non-membrane-bounded organelle | 17 | 4.2x10^–1^ |
| GO-CC | Non-membrane-bounded organelle | 17 | 4.2x10^–1^ |

| Response to metal | | | |
| --- | --- | --- | --- |
| Annotation | Term | Count | *p*-value |
| GO-BP | Response to cadmium ion | 3 | 4.1x10^–2^ |
| GO-BP | Response to metal ion | 4 | 5.2x10^–2^ |
| GO-BP | Response to inorganic substance | 4 | 1.0x10^–1^ |

| Translational regulation | | | |
| --- | --- | --- | --- |
| Annotation | Term | Count | *p*-value |
| GO-BP | Regulation of cellular protein metabolic process | 6 | 1.7x10^–2^ |
| GO-BP | Regulation of translation | 4 | 1.0x10^–1^ |
| GO-BP | Posttranscriptional regulation of gene expression | 6 | 1.7x10^–1^ |

1. Clustering of gene sets were conducted based on the terms of GO-BP，GO-CC，and GO-MF analyses. Most typical terms are shown separately for the stages of *en-pou5f3* induction and the manners of expression changes.
